# Improving CAR-T cell function through a targeted cytokine delivery system utilizing car target-modified extracellular vesicles

**DOI:** 10.1186/s40164-025-00701-z

**Published:** 2025-08-25

**Authors:** Yuanyuan Zhang, Meijuan Huang, Shujia Zhang, Tianjiao Liu, Shanwei Ye, Yuhang Cheng, Yang Cao, Liting Chen, Li Zhu, Xueyan Sun, Kefeng Shen, Qian Xu, Tongjuan Li, Dengju Li, Liang Huang, Wei Mu, Lei Zhao, Jue Wang

**Affiliations:** 1 Department of Hematology, Tongji Hospital, Tongji Medical College, Huazhong University of Science and Technology, Wuhan, China; 2 Immunotherapy Research Center for Hematologic Diseases of Hubei Province, Wuhan, China; 3 Department of Hematology and Oncology, Ningbo No.2 Hospital, Ningbo, Zhejiang Province China; 4 State Key Laboratory of Experimental Hematology, Haihe Laboratory of Cell Ecosystem, Institute of Hematology & Blood Diseases Hospital, National Clinical Research Center for Blood Diseases, Chinese Academy of Medical Science & Peking Union Medical College, Tianjin, China; 5 Tianjin Institutes of Health Science, Tianjin, China

**Keywords:** Cellular immunotherapy, Chimeric antigen receptor T cell, Extracellular vesicles, Interleukin-12

## Abstract

Chimeric antigen receptor (CAR)-T-cell therapy has achieved remarkable clinical success in the treatment of B-cell malignancies; however, its efficacy can be limited by poor T-cell persistence and insufficient antitumor activity in certain cases. Moreover, interleukin-12 (IL-12) is a prominent agent in cancer immunotherapy, but its clinical application is constrained by severe toxicity associated with systemic exposure. In this study, we developed a novel cytokine delivery platform based on CAR target-modified cell-derived extracellular vesicles (EVs) that preferentially bind CAR-T cells to improve CAR-T-cell function. EVs with surface-displayed CD19 and/or IL-12 were successfully generated from HEK-293T cells. Compared with an equivalent concentration of rhIL-12, IL-12 EVs significantly enhanced the effector function of anti-CD19 CAR-T cells in vitro, resulting in increased Interferon-γ (IFN-γ) and TNF-α secretion, cytolytic activity, and T-cell expansion. Additionally, compared with EVs expressing IL-12 alone, EVs co-expressing IL-12 and CD19 (CD19/IL-12 EVs) exhibited superior binding efficiency to CAR-T cells but not to T cells, as indicated by flow cytometry. In xenograft model mice bearing CD19 + Raji tumors, intratumoral injection of CD19/IL-12 EVs resulted in durable antitumor responses and enhanced the in vivo expansion of CAR-T cells, outperforming CD19 EVs, IL-12 EVs and control EVs, without causing systemic toxicity. RNA sequencing (RNA-seq) analysis of CAR-T cells stimulated with EVs suggested that the increased efficacy was driven by IL-12 signaling. These data demonstrate that CAR-targeted modified EVs may serve as targeted cytokine delivery systems for CAR-T cells, offering a safe and effective strategy to augment CAR-T-cell function.

**Supplementary Information:**

The online version contains supplementary material available at 10.1186/s40164-025-00701-z.

## Introduction

Chimeric antigen receptor (CAR)-T-cell therapy has achieved clinical success in the treatment of hematological cancers, especially B-cell malignancies [1, 2]. However, more than half of patients treated with CAR-T-cell therapy fail to achieve long-term remission [3, 4]. Moreover, the treatment of solid tumors with CAR-T-cell therapy has major challenges [5]. Several factors limit the clinical benefits of CAR-T cells, such as insufficient T-cell proliferation, weak T-cell antitumor activity, limited T-cell persistence and the immunosuppressive tumor microenvironment (TME) [6–8]. To overcome these hurdles, many preclinical studies and clinical trials have been conducted to enhance the desirable properties of CAR-T cells in vivo by co-administering cytokines, with IL-12 being a promising candidate for combination immunotherapy. IL-12, a heterodimeric protein consisting of the p40 and p35 subunits, is a proinflammatory cytokine with robust anti-tumor activity [9, 10]. IL-12 is produced mainly by dendritic cells (DCs), B lymphocytes, and macrophages, and can bind to IL-12Rβ1 and IL-12Rβ2 on the surface of T cells [11]. IL-12 plays a crucial role in the differentiation of naïve T cells into T helper type 1 (Th1) cells and the activation of T cells, increasing their proliferation, IFN-γ production and cytotoxic potential [9, 10]. Many studies have demonstrated that IL-12 can enhance the cytokine release and antitumor activity of CAR-T cells and significantly extend their persistence in vivo [12–16].

In the rapidly advancing field of nanomedicine, extracellular vesicles (EVs) have emerged as robust and feasible nanovesicle delivery systems [17]. EVs are membrane particles secreted by almost all cells in an inducible or constitutive manner [18, 19]. Naturally, released EVs can function as important regulators by transporting nucleic acids or proteins to nearby or distal cells. Numerous studies have demonstrated the potential of EVs as nanocarriers for delivering cytokines in vivo to exert effects on target cells in a stable and specific way [20–22]. Moreover, it has been suggested that surface molecules displayed by EVs can interact with receptors on T cells, including CAR-T cells, thereby regulating their functions [23–26]. Therefore, modifying the EV surface with IL-12 may represent a potent strategy to specifically deliver IL-12 to CAR-T cells, thereby enhancing CAR-T-cell function in vivo.

In this study, we aimed to modify EVs with CD19, the most commonly adopted target in CAR-T-cell therapy, to specifically deliver IL-12 to anti-CD19 CAR-T cells.

We isolated EVs displaying CD19 and IL-12 on their membranes (named CD19/IL-12 EVs) from parental cells that artificially and simultaneously expressing CD19 and IL-12. These CD19/IL-12 EVs were tested for their binding affinity and biofunctions with anti-CD19 CAR-T cells both in vitro and in vivo. CD19/IL-12 EVs enhanced the function of CAR-T cells by strengthening their cytotoxic effects and regulating gene expression without inducing systemic toxicity. Our work provides proof-of-principle evidence suggesting that CAR target-modified EVs constitute an effective and safe delivery system for the pleiotropic cytokine IL-12 and a bioenhancer for CAR-T cells.

## Results

### Manufacturing of EVs with functional, surface-displayed IL-12

**Fig. 1:**
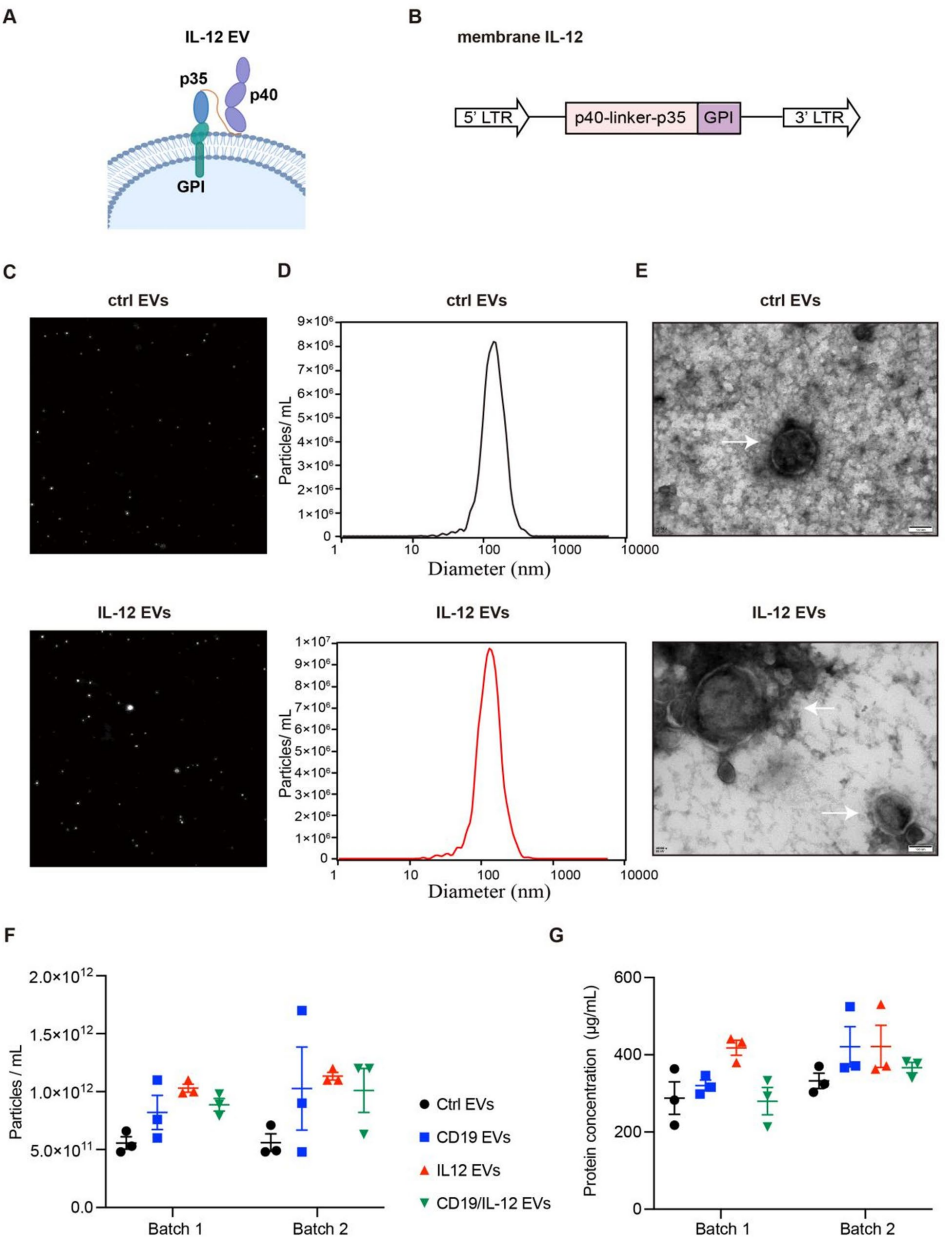
Design and Characterization of control EVs. (**A**) IL-12 EVs were engineered EVs that displayed single-chain IL-12 on their surface through the membrane-anchored structure GPI. (**B**) A schematic diagram of the plasmid used for membrane IL-12 expression. (**C**, **D**) NTA was used to examine the Brownian motion and size distribution of EVs. (**E**) TEM images of EVs. (**F**) Nanoparticle concentration of different types of EVs from two independent production batches was quantified by nanoparticle tracking analysis (NTA). Each batch included three biological replicates. (**G**) Protein concentration of the same EV samples was measured using the BCA protein assay (independent experiments with *n* = 3)

**Fig. 2:**
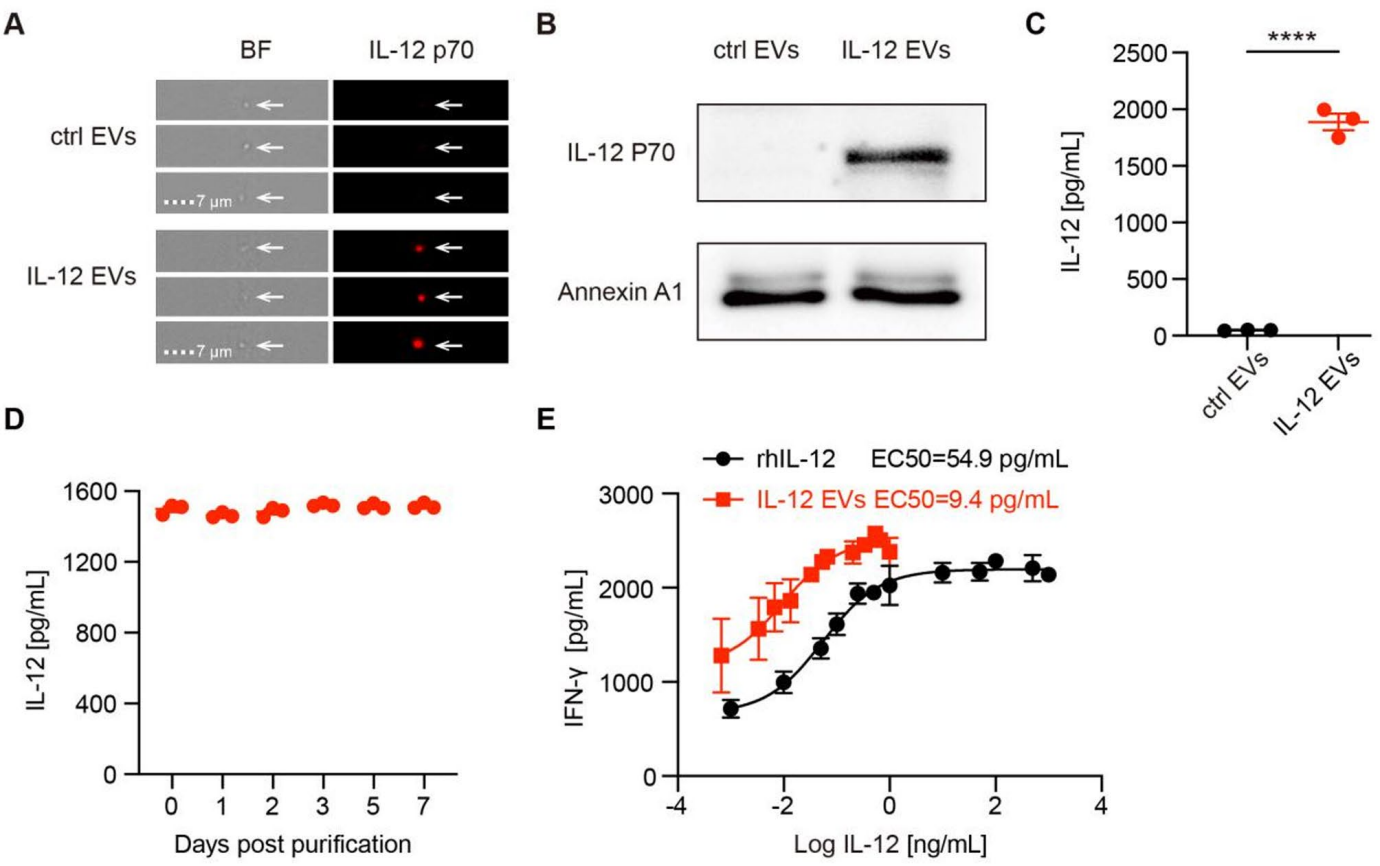
Manufacture of EVs with functional, surface-displayed IL-12. (**A**) Representative images of IL-12 EVs and control EVs characterized by flow cytometry (independent experiments with *n* = 3). (**B**) Annexin A1 and IL-12 p70 expression in EVs was detected by western blotting (independent experiments with *n* = 3). (**C**) The IL-12 concentrations in EVs were quantified via ELISA, and the mean IL-12 concentration of IL-12 EVs was 1887 pg/mL (independent experiments with *n* = 3). **D**) IL-12 EVs were stored at − 80 °C, and their concentration was determined by ELISA analysis at different time points (independent experiments with *n* = 3). (**E**) Representative dose‒response curves of IFN-γ secretion from T cells (cell density: 1*10^6^/mL) after treatment with doses of rhIL-12 or IL-12 EVs. The EC50 values derived from the graphs were 54.9 pg/mL and 9.4 pg/mL, respectively (independent experiments with *n* = 3). Data are presented as mean ± SEM. Statistical analysis was performed using unpaired t test. ****p < 0.0001

To obtain IL-12 EVs, we established a HEK293T cell line that expresses IL-12 on the cell surface through the membrane-anchored structure glycosylphosphatidylinositol (GPI) (Fig. 1A and B). The flow cytometry results indicated that IL-12 was highly expressed on the membrane surface of the engineered HEK293T cells (Figure S1A). Control EVs and IL-12 EVs were examined via nanoparticle tracking analysis (NTA) and transmission electron microscopy (TEM), and their Brownian motion, size distribution and morphology were monitored. There was no difference in the above parameters between the two types of EVs (Fig. 1C, D, E). No significant batch effect was observed in particle density and protein concentration (Fig. 1F, G). Imaging flow cytometry was used to identify IL-12 expression on the membrane surface of IL-12 EVs (Fig. 2A). Western blotting confirmed that Annexin A1 was expressed in both types of EVs and that IL-12 p70 was expressed only in IL-12 EVs (Fig. 2B, Figure S2A). The IL-12 concentration in EVs was determined via ELISA (Fig. 2C). To confirm the stability of IL-12 EVs, we found that there was no difference in the IL-12 concentration among EVs stored at − 80 °C for different periods of time (Fig. 2D). Furthermore, IFN-γ secretion from T cells after treatment with different concentrations of rhIL-12 or IL-12 EVs was tested to determine whether IL-12 EVs can stimulate T-cell activation. We found that the EC50 value of IL-12 EVs was approximately 9.4 pg/mL (4.84 × 10^9^ EV particles/mL), which was lower than that of rhIL-12 (Fig. 2E).

### IL-12 EVs enhance the function of CAR-T cells and NK cells

**Fig. 3:**
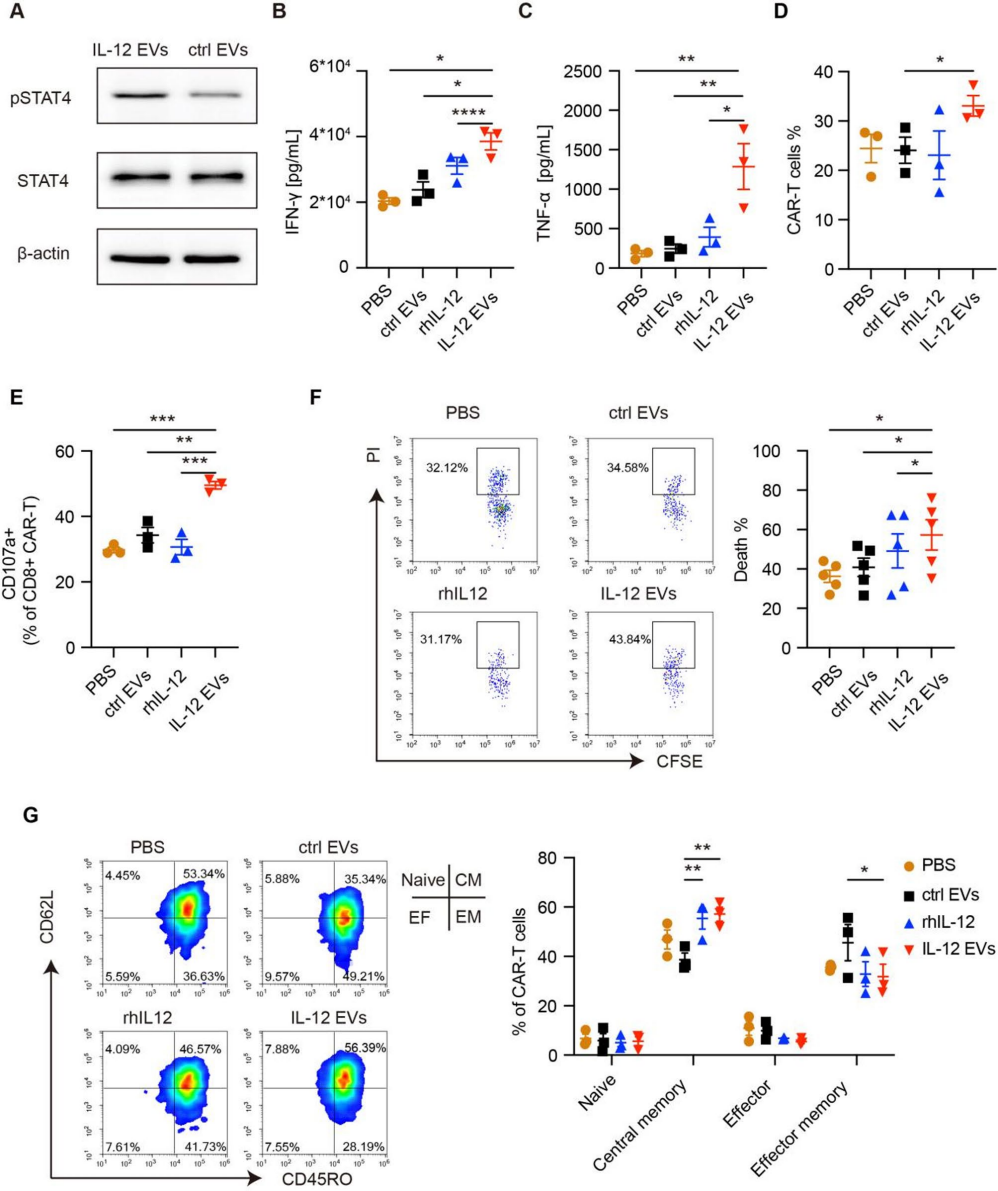
IL-12 EVs enhance the function of CAR-T cells. (**A**) CAR-T cells were treated with control EVs or IL-12 EVs for 30 min, and the phosphorylation of STAT4 was analyzed by western blotting. Experiments are representative of 3 independent repeats with similar results. (B-G) CAR-T cells (cell density: 1*10^6^/mL) were mixed with 5 × 10^4^ Raji cells at an effector-to-target ratio of 1:1 and treated with PBS, control EVs, rhIL-12, and IL-12 EVs repectively. Protein concentration of EVs is 167 µg/mL, and rhIL-12 concentration is 667 pg/mL. (**B**, **C**) Cytokine secretion by CAR-T cells was detected by ELISA after 24 h of coculture (*n* = 3 donors). (**D**) The proportion of CAR-T cells was analyzed by flow cytometry after 72 h of coculture (*n* = 3 donors). (**E**) CD107a expression in CD8 + CAR-T cells was detected by flow cytometry (*n* = 3 donors). (**F**) Raji cell death was determined using PI (BD Pharmingen) and analyzed by using flow cytometry after 24 h (*n* = 5 donors). (**G**) Subsets were detected via flow cytometry in CAR-T cells after 7 days of treatment (*n* = 3 donors). Data are presented as mean ± SEM. Statistical analysis was performed using one-way or two-way ANOVA. *p < 0.05, **p < 0.01, ***p < 0.001, ****p < 0.0001

Since IL-12 can activate STAT phosphorylation in T cells, we investigated the effects of IL-12 EVs on STAT4 phosphorylation in anti-CD19 CAR-T cells in vitro. Western blotting confirmed that the level of phosphorylated STAT4 in CAR-T cells after IL-12 EV treatment was greater than that in the control cells (Fig. 3A, Figure S2B). To verify that IL-12 EVs enhance CAR-T-cell function, we co-incubated 5 × 10^4^ anti-CD19 CAR-T cells with Raji cells at an effector-to-target ratio of 1:1 and treated them with PBS, control EVs, rhIL-12, or IL-12 EVs. EVs were quantified on the basis of total protein content, with a uniform concentration of 167 µg protein per milliliter for each formulation. After 24 h of coculture, IFN-γ and TNF-α secretion by CAR-T cells was significantly greater in the IL-12 EV group than in the control EV group and greater than that in the rhIL-12 group at the same estimated IL-12 concentration of 667 pg/mL (Fig. 3B and C). Similarly, the proportion of CAR-T cells in the IL-12 EV group was also the highest among the above four groups (Fig. 3D). For analysis of the surface expression of CD107a, CAR-T cells exposed to IL-12 EVs exhibited more pronounced degranulation than the other groups did (Fig. 3E). Moreover, IL-12 EVs significantly increased the antitumor activity of CAR-T cells against Raji cells (Fig. 3F). When K562 cells without CD19 expression were used as target cells, CAR T cells in four groups exhibited very low CD107a expression, and no significant tumor cell death was observed (Figure S3A, B). These findings demonstrated that IL-12 EVs promoted antigen-specific cytotoxicity of CAR-T cells and no off-target toxicity was observed. Next, we analyzed the cell subsets and immune checkpoint expression of CAR-T cells treated with IL-12 EVs. IL-12 EVs induced an increase in the number of CAR-T cells with the central-memory phenotype and a decrease in the number of CAR-T cells with the effector-memory phenotype (Fig. 3G). Interestingly, IL-12 EVs induced higher levels of Lymphocyte Activation Gene 3 (LAG-3) expression than the other EVs did, but there was no significant difference in Programmed cell death-1 (PD-1) expression (Figure S3C). Taken together, these data demonstrated that IL-12 EVs promoted CAR-T-cell expansion and functional maturation.

Natural killer (NK) cells are known to express IL-12 receptor. To test whether IL-12 EVs can stimulate NK cells, we cocultured human NK cells with different EVs and assessed intracellular pSTAT4 (Y693) levels and IFN-γproduction of NK cells. Treatment with IL-12 EVs resulted in higher levels of phosphorylated STAT4 in NK cells compared to both control EVs and rhIL-12 group at equivalent estimated IL-12 concentration (Figure S4A). Moreover, IFN-γ secretion by NK cells was significantly elevated in the IL-12 EV group relative to both the control EV group and the rhIL-12 group (Figure S4B). These results indicated that IL-12 EVs not only enhance T cell function, but can also stimulate NK cells. Therefore, more precise delivery methods are needed for the in vivo delivery of IL-12 EVs to avoid potential adverse effects.

### Targeted delivery of cytokines to enhance CAR-T cell function

Although IL-12 has been shown to enhance the function of CAR-T cells, systemic administration of IL-12 at therapeutic doses triggers severe inflammatory reactions and uncontrolled toxicity [27], thus limiting its clinical application in combination with CAR-T-cell immunotherapy. Previously, we demonstrated that EVs presenting CAR-specific antigens can be preferentially absorbed by CAR-T cells and regulate their function [28]. Therefore, we utilized IL-12 EVs with surface antigen expression as a strategy for targeted IL-12 delivery to CAR-T cells, thereby specifically enhancing CAR-T-cell function in vivo.

**Fig. 4:**
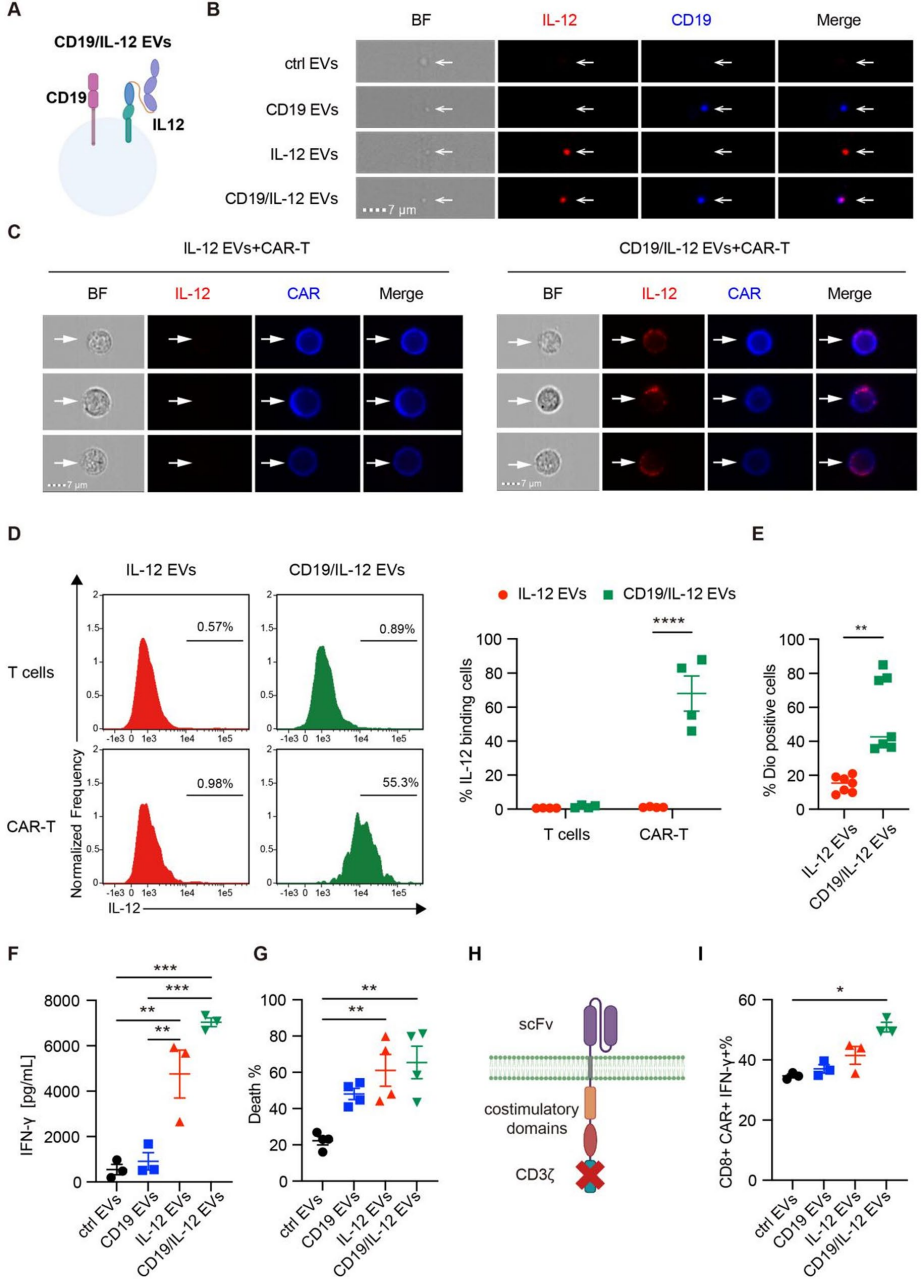
CD19/IL-12 EVs specifically bind to anti-CD19 CAR-T cells and increase their antitumor activity. (**A**) A schematic diagram of CD19/IL-12 EVs. (**B**) Representative images of control EVs, CD19 EVs, IL-12 EVs and CD19/IL-12 EVs characterized by flow cytometry (independent experiments with *n* = 3). (**C**) IL-12 EVs or CD19/IL-12 EVs were added to 1 × 10^6^ CAR-T cells for 45 min, and representative images of CAR-T cells decorated with EVs were characterized by flow cytometry (independent experiments with *n* = 3). (**D**) CAR-T cells were incubated with IL-12 EVs or CD19/IL-12 EVs for 45 min, and the efficiency of EV-binding CAR-T cells and non-CAR-T cells was revealed by flow cytometry (*n* = 4 donors). (**E**) CAR-T cells were treated with DiO-labeled IL-12 EVs or CD19/IL-12 EVs for 4 h, and the cellular internalization of DiO-labeled EVs by CAR-T cells was analyzed via flow cytometry (*n* = 7 donors). (F, G) CAR-T cells were mixed with Raji cells at an effector-to-target ratio of 1:1 and treated with control EVs, CD19 EVs, IL-12 EVs or CD19/IL-12 EVs for 24 h. (**F**) IFN-γ secretion by CAR-T cells was measured via CBA. (**G**) Raji cell death was determined using PI (BD Pharmingen) and analyzed using flow cytometry (*n* = 4 donors). (**H**) A schematic diagram of the ΔCAR plasmid, namely, removing CD3ζ from the normal CAR structure. (**I**) ΔCAR-T cells were treated with control EVs, CD19 EVs, IL-12 EVs or CD19/IL-12 EVs for 8 h, and the intracellular IFN-γ levels in the CD8 + CAR-T cells were detected via flow cytometry (independent experiments with *n* = 3). Data are presented as mean ± SEM. Statistical analysis was performed using one-way or two-way ANOVA. *p < 0.05, **p < 0.01, ***P*p* < 0.001, ****p < 0.0001.Protein concentration of each EV is 167 µg/mL

First, we established a HEK293T cell line co-expressing IL-12 and CD19 on the cell surface to obtain CD19/IL12 EVs (Fig. 4A). Flow cytometry indicated that both IL-12 and CD19 were highly expressed on the membrane surface of the constructed HEK293T cells (Figure S5A). Notably, there was no difference in Brownian motion or size distribution between CD19 EVs and CD19/IL-12 EVs (Figure S5B, S5C). As shown in Figure S5D, there was no significant difference in the cell viability of CAR-T cells among the control EVs, IL-12 EVs, CD19/IL-12 EVs, and rhIL-12 groups. Similarly, tumor cells cultured in the absence of T cells exhibited comparable viability across all EV treatment groups. These findings demonstrate that neither the EVs nor rhIL-12 affect viability of T cells or tumor cells. Imaging flow cytometry was used to confirm that IL-12 and CD19 were expressed simultaneously on the surface of the CD19/IL-12 EVs (Fig. 4B). Next, to test the delivery efficiency of CD19/IL-12 EVs in vitro, we incubated the same dose of IL-12 EVs or CD19/IL-12 EVs with 1 × 10^6^ anti-CD19 CAR-T cells for 45 min. Flow cytometry analysis revealed that CAR-T cells bound more IL-12 molecules in the CD19/IL-12 EV group than in the IL-12 EV group (Fig. 4C). CAR T cells in CD19/IL-12 EVs group exhibited higher CD107a expression than control EVs group and IL-12 EVs group (Figure S5E). Moreover, a stronger IL-12 p70 signal was observed only in CAR-T cells and not in T cells (Fig. 4D), indicating that the increased IL-12 expression was due to the interaction between CD19 and the CAR. Furthermore, to verify whether the elevated IL-12 expression in CAR-T cells was the result of exogenous delivery from EVs or CAR-induced downstream cellular signaling activation, we treated 3 × 10^5^ CAR-T cells with DiO-labeled IL-12 EVs or CD19/IL-12 EVs for 4 h and evaluated the cellular internalization of DiO-labeled EVs via flow cytometry. As a result, there were significantly more positive CAR-T cells labeled with Dio in the CD19/IL-12 EV group than in the IL-12 EV group (Fig. 4E), suggesting that CAR-dependent antigen/receptor binding facilitated the targeted delivery of membrane-bound molecules of EVs, including IL-12.

Then, we mixed CAR-T cells with 5 × 10^4^ Raji cells at an effector-to-target ratio of 1:1 and treated them with control EVs, CD19 EVs, IL-12 EVs or CD19/IL-12 EVs for 24 h to determine whether the IL-12 delivered by EVs could exert bioactive effects. Compared with control EVs, both IL-12 EVs and CD19/IL-12 EVs were able to induce greater IFN-γ secretion and cellular toxicity (Fig. 4F, G). To analyze the long-term effects of EV treatments, 3 × 10⁵ CAR-T cells were co-cultured with Raji cells, and treated with PBS, control EVs, CD19 EVs, IL-12 EVs, or CD19/IL-12 EVs over three sequential rounds (Figure S6A). After each round, CAR-T cell subsets and immune checkpoint expression were analyzed. Following three rounds of EV treatments, IL-12 EVs and CD19/IL-12 EVs treatments initially led to a reduction in the proportion of CAR-T cells with a central-memory phenotype after the first round and second round, but induced an increase after the third round. In parallel, one or two rounds of IL-12 EV or CD19/IL-12 EV treatment promoted an increase in the proportion of CAR-T cells with an effector-memory phenotype, which subsequently declined after the third round (Figure S6B). This dynamic shift suggests that IL-12 EVs or CD19/IL-12 EVs may initially enhance effector function, but with prolonged stimulation, they may also support the memory pool, potentially improving the durability of CAR-T cell responses. Moreover, IL-12 EVs and CD19/IL-12 EVs consistently induced higher expression levels of LAG-3 and PD-1 compared to other EV groups across all three rounds of stimulation. In contrast, TIM-3 expression remained largely unchanged, except for a transient increase after the second round. Interestingly, IL-12 EVs and CD19/IL-12 EVs induced lower levels of TIGIT expression than the other EVs (Figure S6C).

Furthermore, to rule out the possible effects of anti-CD19 CAR-induced T-cell activation with CD19/IL-12 EVs, we generated CAR-T cells using a CAR structure lacking the CD3ζ signal sequence (referred to as the ΔCAR hereafter) (Fig. 4H). Therefore, the influence of the targeted delivery of IL-12 by CD19/IL-12 EVs could be observed independently of CAR-induced T-cell signaling. Interestingly, IFN-γ secretion by CD8 + ΔCAR-T cells was significantly greater in the CD19/IL-12 EV group than in the control, CD19 or IL-12 EV groups (Fig. 4I). Taken together, these findings indicate that CD19/IL-12 EVs preferentially bind to anti-CD19 CAR-T cells and enhance their antitumor effects in vitro.

**Fig. 5:**
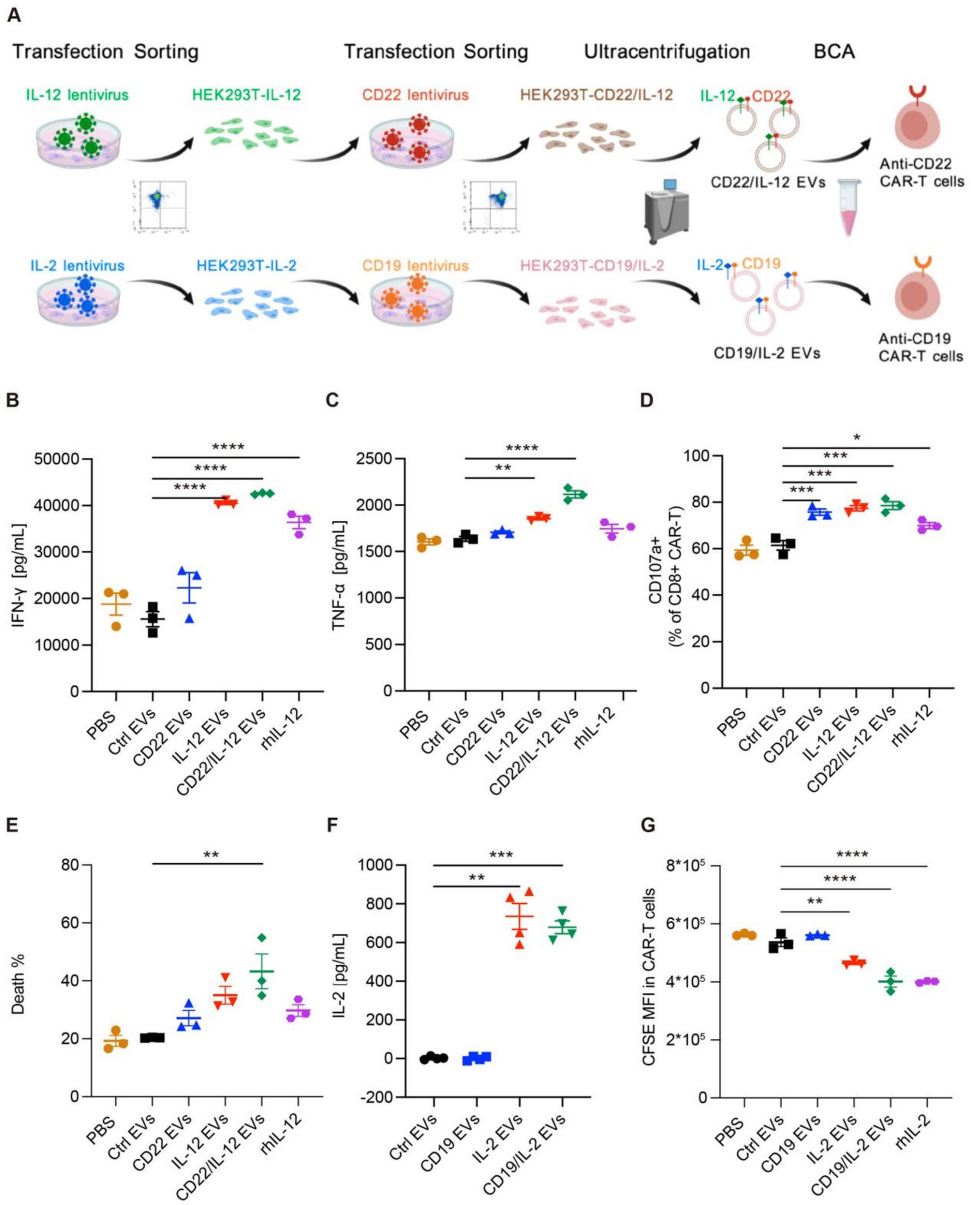
Targeted delivery EV to CD22 CAR-T cells and targeted delivery IL-2 EV to CD19 CAR-T cells. (**A**) Schematic experimental workflows of EV production and quality control node. (**B**-**D**) 3 × 10⁵ CAR-T cells (cell density: 1*10^6^/mL) were mixed with Raji cells at an effector-to-target ratio of 1:1 and treated with PBS, control EVs, rhIL-12 and IL-12 EVs respectively (*n* = 3 donors). (**B**) Cytokine secretion by CAR-T cells was detected by ELISA after 24 h of coculture. (**C**) CD107a expression in CD8 + CAR-T cells was detected by flow cytometry. (**D**) Raji cell and k562 cell death were determined using PI (BD Pharmingen) and analyzed by using flow cytometry after 24 h. (**E**) Subsets were detected via flow cytometry in CAR-T cells after 7 days of treatment. (**F**) Quantification of IL-2 concentration in EVs by ELISA (independent experiments with *n* = 3). Mean ± SEM. (**G**) CD19 CART cells labeled with CFSE were cocultured with various types of EVs for 96 h (*n* = 3 donors). *p < 0.05. Protein concentration of EVs is 167 µg/mL, and rhIL-12 concentration is 667 pg/mL

To assess whether this targeted EV delivery system can be applied to CAR-T cells specific for other antigen, we generated HEK293T cell lines over-expressing CD22 alone or HEK293T co-expressing CD22 and IL12, as shown in the experimental workflow of Fig. 5A. Flow cytometry analysis confirmed high surface expression of CD22 or co-expression of CD22 and IL-12 on the engineered HEK293T cells (Figure S7A). We then co-cultured CD22 CAR-T cells with Raji cells and treated them with control EVs, CD22 EVs, IL-12 EVs, CD22/IL-12 EVs, or rhIL-12 to evaluate the bioactivity of IL-12 delivered by EVs. Compared to control EVs, both IL-12 EVs and CD22/IL-12 EVs were able to enhance IFN-γ and TNF-α secretion and cellular toxicity in CD22 CAR-T cells (Fig. 5B and C). Notably, CD22/IL-12 EVs induced significantly higher CD107a expression and antitumor activity than IL-12 EVs (Fig. 5D and E).

To investigate whether the EVs can be engineered to incorporate other cytokines, we generated HEK293T cell lines over-expressing a modified IL-2 variant [29], as well as HEK293T cells co-expressing CD19 and the modified IL-2. Surface expression of IL-2 in the engineered HEK293T cells was confirmed by flow cytometry and ELISA, while co-expression of CD19 and IL-2 was validated by flow cytometry (Fig. 5F, Figure S7B). CFSE-based proliferation assays demonstrated that IL-2 EVs and CD19/IL-2 EVs derived from these engineered HEK293T cells significantly enhanced CAR-T cell expansion compared to control EVs (Fig. 5G).

### CD19/IL-12 EVs increase CAR-T-cell proliferation and antitumor activity in vivo

**Fig. 6:**
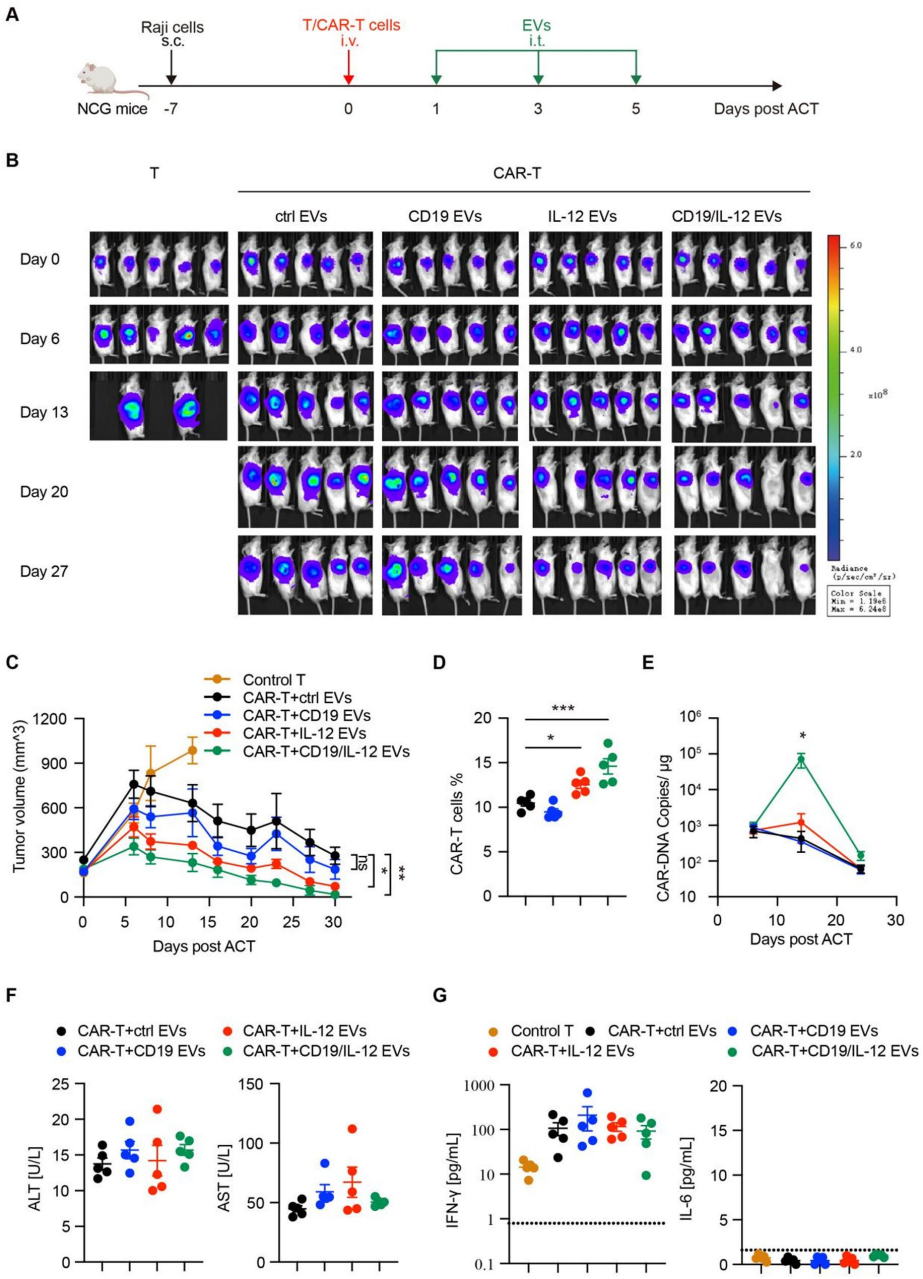
Intratumoral administration of CD19/IL-12 EVs increases CAR-T-cell proliferation and antitumor activity in vivo. (**A**) Experimental scheme of the in vivo antitumor experiment (*n* = 5 mice per group, 1 × 10^6^ CAR-T cells with CAR transduction efficiency of 15% per mouse, or 6.67 × 10^6^ untransduced T cells per mouse in control group). (**B**) Tumor growth and stage were monitored via bioluminescence imaging (BLI). (**C**) Tumor growth curves of Raji tumor-bearing mice, and the tumor volumes were calculated using the following formula: volume = (length×width^2^)/2. (**D**) The proportion of CAR-T cells in the peripheral blood was analyzed via flow cytometry on day 9 after adoptive T-cell transfer (ACT). (**E**) The cellular kinetics of CAR-T cells transferred into mice were determined by quantifying the number of CAR transgene copies in the peripheral blood via droplet digital PCR. (**F**) Hepatic function (Alanine Aminotransferase, ALT; Aspartate Aminotransferase, AST) of the mice was detected by chemical tests on day 14 after ACT. (**G**) The serum levels of cytokines (IFN-γ and IL-6) in the mice were quantified by using cytometric bead array (CBA) kit on day 9 after ACT. A dashed line is indicative of the lower detection limits. Data are presented as mean ± SEM. Statistical analysis was performed using one-way ANOVA and log-rank (Mantel‒Cox) test for animal survival. *p < 0.05, **p < 0.01, ***p < 0.001 (data representative of two independent experiments)

To explore the impact of CD19/IL-12 EVs on CAR-T cells in vivo, xenograft model mice bearing CD19 + Raji tumors were infused with CAR-T cells or control T cells (Fig. 6A). We administered control EVs, CD19 EVs, IL-12 EVs or CD19/IL-12 EVs to tumor-bearing xenograft mice via intratumoral injection to reduce potential systemic toxicity. Compared with T cells, CAR-T cells clearly controlled the tumor burden in xenograft model mice within 30 days after infusion. In the mice treated with CAR-T cells and intratumorally injected EVs, the tumor burden in the CD19/IL-12 EV group was the lowest among all the groups, suggesting that CAR-T cells in combination with CD19/IL-12 EVs achieved the best tumor control (Fig. 6B, C). Moreover, compared with other types of EVs, CD19/IL-12 EVs induced outstanding CAR-T-cell amplification, as indicated by flow cytometry and droplet digital PCR (Fig. 6D, E). Thus, CD19/IL-12 EVs could promote the expansion of transferred CAR-T cells and enhance their antitumor effects in vivo.

Mice treated with different EVs were normal in appearance and displayed regular weight gain over time (Figure S8A). EV delivery of IL-12 in vivo may cause additional side effects, such as hepatic dysfunction and severe cytokine release syndrome (CRS); however, hepatic and renal function were monitored, and no significant differences were detected across all the groups of mice (Fig. 6F, S8B). The concentrations of cytokines, including IFN-γ, IL-6, TNF-α, IL-2, IL-12, and IL-4, in mouse serum were also examined. We observed no obvious differences in the above cytokines across groups except for a slight increase in the IL-6 level in the CD19/IL-12 EV group, but the difference was not statistically significant (Fig. 6G, S8C). The physiological structure of the vital organs of the mice was normal (Figure S8D). Taken together, intratumoral administration of CD19/IL-12 EVs increased CAR-T cell proliferation and antitumor activity in vivo without additional adverse events.

### Transcriptomic analyses of CAR-T cells treated with IL-12 EVs and CD19/IL-12 EVs

**Fig. 7:**
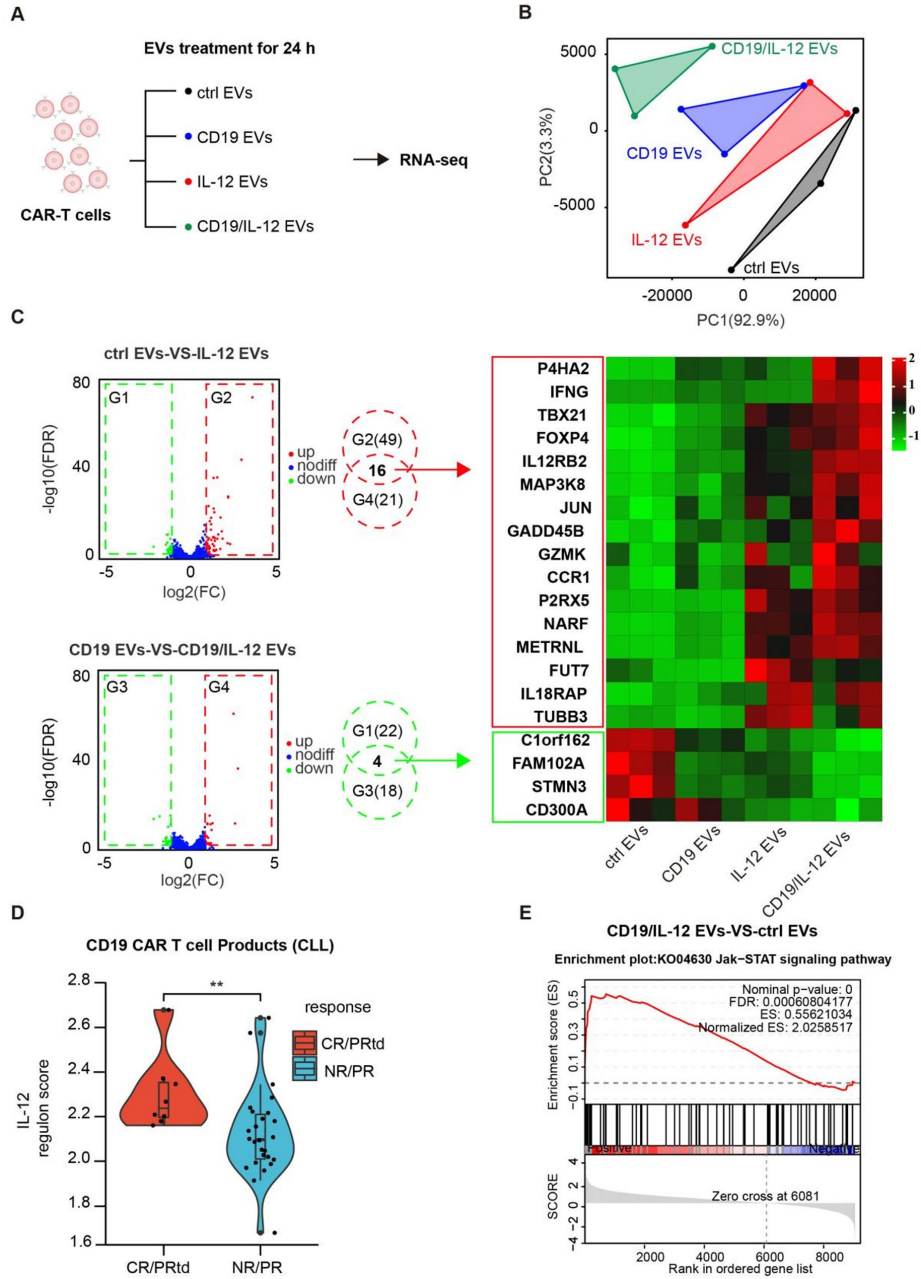
Effects of IL-12 EVs and CD19/IL-12 EVs on the transcriptome of CAR-T cells. (**A**) A schematic view of the study design. CAR-T cells treated with different EVs (protein concentration: 167 µg/mL). (**B**) PCA of the RNA-Seq data. Points represent each sample. The samples in one group are indicated by the same color. Points that are closer together are more similar in terms of gene expression patterns (*n* = 3 donors). (**C**) Genes that shared the same expression pattern of control EVs versus IL-12 EVs and CD19 EVs versus CD19/IL-12 EVs were selected (left), and the expression patterns are listed on the right. (**D**) To evaluate the impact of IL-12-regulated genes on the clinical response, we performed a ssGSEA of IL-12 gene signatures from the RNA-seq data of CAR-stimulated anti-CD19 CAR-T-cell products from 34 CLL patients (complete responders (CRs), *n* = 5; partial responders with transformed disease (PRTDs), *n* = 3; partial responders (PRs), *n* = 5; nonresponders (NRs), *n* = 21). IL12 regulon ssGSEA for patient outcomes in (**D**). (**E**) GSEA plot of the JAK-STAT signaling pathway in control EVs versus CD19/IL-12 EVs. The normalized enrichment score (NES) and statistical significance/false discovery rate (FDR) Q value are indicated. *p < 0.05, **p < 0.01, ***p < 0.001

To confirm that the enhanced CAR-T-cell performance was IL-12-specific and explore the underlying molecular mechanisms of CD19/IL-12 EVs, we performed RNA-seq on CAR-T cells incubated with control EVs, CD19 EVs, IL-12 EVs or CD19/IL-12 EVs (Fig. 7A). Principal component analysis (PCA) revealed a clear separation between the group treated with CD19/IL-12 EVs and the groups treated with other EVs, indicating that CD19/IL-12 EVs uniquely regulate the transcriptome (Fig. 7B).

A total of 90 differentially expressed genes (DEGs) were detected between either control EVs-versus-IL-12-EVs or between CD19-EVs-versus-CD19/IL-12-EVs, including 54 upregulated and 36 downregulated genes. To identify key genes related to IL-12-mediated regulation of CAR-T-cell function, we compared the DEGs between the control EV group and the IL-12 EV group, as well as the DEGs between the CD19 EV group and the CD19/IL-12 EV group, which represented the IL-12-induced transcriptome alterations in both the resting and active conditions of CAR-T cells in the absence or presence of the CD19 antigen. The resulting DEGs were compared, and 16 genes were upregulated and 4 genes were downregulated in both the IL-12 EV and CD19/IL-12 EV groups. Notably, classic IL-12 downstream targets in CAR-T cells, such as IFNG, IL12RB2, and IL18RAP, were identified as expected. Interestingly, we also discovered several previously unreported genes that possibly regulate CAR-T-cell function through IL-12, such as JUN and GZMK (Fig. 7C). GO and KEGG pathway analyses revealed that those DEGs were significantly enriched in positive regulation of the immune system, alpha-beta T-cell activation, and Th1 and Th2 cell differentiation pathways (Supplemental Fig. S9A and Fig. S9B.) Despite these differences, we sought to evaluate whether the 16 upregulated genes were clinically relevant in terms of CAR-T-cell efficacy. We assessed the expression levels of these genes from independent and publicly accessible clinical cohort data previously reported by Fraietta and colleagues [3]. Indeed, our IL-12 regulon score was positively associated with favorable clinical response in patients with chronic lymphocytic leukemia receiving anti-CD19 CAR-T-cell therapy (*p* = 0.007919; Fig. 7D).

Compared with those in control EVs, genes involved in the JAK/STAT, TNF and cytokine-cytokine receptor interaction signaling pathways were significantly enriched in CD19/IL-12 EVs (Fig. 5E, S9C).

### Discussion and conclusion

**Fig. 8:**
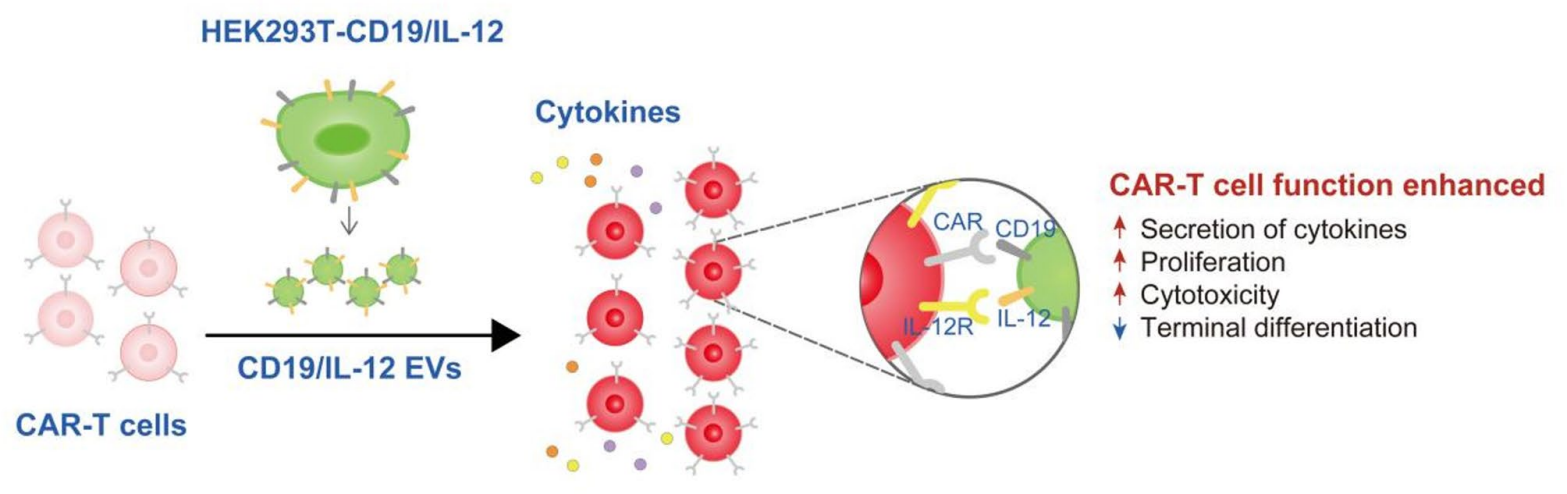
A schematic model of the effect of CD19/IL-12 EVs on CAR-T-cell function. EVs secreted by engineered HEK293T cells stably and simultaneously expressing CD19 and IL-12 were administered to anti-CD19 CAR-T cells, causing specific marked increases in CAR-T-cell expansion, cytokine secretion and cytotoxicity, with limited terminal differentiation

In this study, we generated modified EVs to deliver cytokines to CAR-T cells and enhance their function, which led to the secretion of cytokines, increased cell proliferation, increased cytotoxic ability and restricted terminal differentiation (Fig. 8). Our study provides insights into a targeted cytokine delivery system for CAR-T cells and offers a safe and effective strategy to synergistically improve CAR-T cell function.

In addition to inadequate tumor targeting and immunosuppressive factors that hinder CAR-T cell efficacy, the signals generated by current CAR constructs may be insufficient to fully unleash the antitumor capabilities of T-cells [6–8]. While second-generation CARs provide the first and second signals for T-cell activation, many studies have explored adding a third signal, such as IL-7, CCL 19 or IL-15 [30, 31]. Given the potent effects of IL-12 on CAR-T cells, the combination of CAR-T cells with IL-12 is particularly attractive. Because CAR-T cells continuously produce IL-12 during expansion, conditional or inducible release of IL-12 is preferable to prevent possible treatment-related toxicity compared with constitutive expression [32, 33]. Exogenous IL-12 can be delivered via oncolytic viruses, nanochaperones, or mRNAs [14, 34, 35], and membrane-bound approaches have been used to limit IL-12 distribution and enhance immune-modulating efficacy [32, 33, 36]. Notably, exosome-loaded cytokines exhibit stronger biological activity than equal amounts of recombinant proteins [37]. Consistent with these observations, our work demonstrated that glycosylphosphatidylinositol (GPI)-anchored IL-12, presented in the solid phase via EVs, enhances the antitumor effect of CAR-T cells at a relatively lower working concentration compared with recombinant human IL-12. Notably, a previous study reported that displaying GPI-anchored proteins on EVs increases EV binding to target cells, but does not increase their uptake by these cells [38]. Other forms of membrane-anchoring strategies may also achieve similar effects.

Since EVs can be engineered to deliver multiple signals, we co-expressed CD19 and IL-12 on the EV membrane to simultaneously provide the first and third signals to CAR-T cells. We also tested other receptor/cytokine pairs, such as CD22/IL-2, supporting the modularity of this strategy. Future optimization may involve fine-tuning the ratio of targeting versus stimulatory molecules. However, as the current CD19/IL-12 EVs lack a logic-gated design, off-target interactions are possible, such as presentation of CD19 molecules to IL-12Rβ-positive non-CAR-T cells, especially in a systemic context. This underscores the need for careful ligand and payload selection, and the potential of programmable protein ligation systems to enhance targeting specificity [39].

Although IL-12 has shown greater potential than other clinically approved adjuvant cytokines in enhancing CAR-T function [40], its systemic administration at therapeutic can induce severe inflammatory reactions and overwhelming toxicity [27], including weight loss and reduced survival rates in mice [41], as well as hepatic dysfunction, high fever, and hemodynamic instability in humans [42]. Its short half-life in vivo further limits clinical utility [43]. Previous clinical studies have demonstrated that systemic administration of recombinant IL-12 (rhIL-12) causes hepatotoxicity and pulmonary edema—organs in which EVs are known to accumulate following intravenous injection. While EVs display lower immune clearance upon systemic administration in rodents [44], intravenous delivery of IL-12-loaded EVs may still cause systemic toxicity. In our study, we administered CD19/IL-12 EVs intratumorally to maximize IL-12 exposure to the TME while minimizing systemic toxicity, aligning with current clinical trials where intratumoral IL-12 delivery via immunocytokine fusions, plasmids, viruses, cells or mRNAs has achieved robust antitumor immunity with reduced adverse events [45]. For systemic applications, further optimization of targeted EV delivery systems, such as incorporating CD47 ‘don’t eat me’ signal, is warranted. Nevertherless, direct in vivo comparisons of efficacy and toxicity between CD19/IL-12 EV and rhIL-12 are critical for clinical translation.

The intrinsic heterogeneity of EVs, including the diverse distribution of surface and internal molecules, remains a key challenge for clinical translation. Emerging technologies such as Multiparametric Single-Vesicle Flow Cytometry [46], single-molecule localization microscopy, and resistive pulse sensing [47] may facilitate detailed EV cargo profiling. Since naturally shed EVs contain complex bioactive content, we performed RNA-seq to examine whether the enhanced CAR-T cell activity was specifically driven by exogenous IL-12 delivered via EVs. We identified not only classic IL-12 downstream targets but also previously unreported genes potentially involved in IL-12-mediated CAR-T regulation, such as CCR1 and P2RX5. Notably, c-Jun has been shown to counteract CAR-T cell exhaustion and improve therapeutic efficacy [48]. Furthermore, the IL-12 regulon, genes upregulated in anti-CD19 CAR-T cells upon exogenous IL-12 stimulation, was associated with better clinical responses in chronic lymphocytic leukemia (CLL) patients treated with anti-CD19 CAR-T cells. These findings support further evaluation of EV-mediated IL-12 delivery in clinical settings and suggest that the IL-12 regulon could be a key mediator of therapeutic benefit as well as a predictive biomarker of CAR-T cell efficacy.

In summary, this study provides the first evidence that CAR target-modified EVs can serve as a localized and targeted delivery system for the pleiotropic cytokine IL-12, offering a potentially effective and safe adjuvant approach to enhance CAR-T cell therapy.

## Methods

### Cell lines and cell culture

The HEK293T (human embryonic kidney cells), Raji (Burkitt lymphoma cells), and K562 (chronic myelogenous leukemia cells) cell lines were obtained from the American Type Culture Collection (ATCC, USA). For in vivo studies, Raji cells were transduced with luciferase lentivirus and sorted to obtain 100% purity. Raji and K562 cells were cultivated in RPMI-1640 medium (Gibco, USA) supplemented with 10% FBS (Gibco, USA) and 1% glutamine (Gibco, USA), whereas HEK293T cells were cultured in DMEM (Gibco, USA) supplemented with 10% FBS and 1% glutamine (Gibco, USA). All the cell cultures were cultured at 37 °C in a humidified incubator with 5% CO_2_. The viability of all cells, including T cells, CAR-T cells, and tumor cells, was assessed using Trypan Blue staining before the experiments.

### Plasmid preparation

The lentiviral vector encoding CD19, pCDH-CMV-CD19, was obtained from GeneCopoeia. The plasmid encoding human CD22 was purchased from GeneChem (Shanghai, China). The CD19 and CD22 cDNA were amplified and cloned into a lentiviral expression vector provided by GenScript. To express IL-12, a plasmid encoding membrane-anchored IL-12 was constructed by linking the human IL12 subunit p35 and p40 sequences via a flexible linker coupled to a GPI-anchor signal sequence. Similarly, membrane-anchored super IL-2 was constructed by linking the human super IL-2 sequence to a GPI-anchor signal sequence through a flexible linke. These DNA fragments were synthesized, cloned, and inserted into the same lentiviral expression vector provided by GenScript.

The anti-CD19 CAR and anti-CD22 CAR lentiviral plasmid were constructed as described previously [49]. For laboratory verification, the anti-CD19 CAR gene was linked to a truncated sequence of epidermal growth factor receptor (EGFRt) via the T2A sequence [50]. The CD3ζ-deficient anti-CD19 CAR lentiviral plasmid was generated by removing the CD3ζ signaling domain from the aforementioned structures.

### Lentivirus preparation and transfection

Lentiviral particles were produced by transient transfection of HEK293T cells with packaging vectors of psPAX2 and pMD2.G and lentiviral expression vectors containing the target plasmids at a 1:1 ratio using PEI reagents. After 48 h of transfection, the viral supernatants were collected and centrifuged at 4 °C for 30 min to remove debris. The supernatants were passed through a 0.45 μm Thermo Scientific filter, and the viral particles were subsequently concentrated via centrifugation at 4 °C and 30,000 × g for 150 min (Avanti J-26 S XPI high-performance centrifuge, Beckman Coulter). To determine lentiviral titer, HEK293T cells were transduced with serial dilutions of concentrated lentiviral particles. 48 h post-transduction, genomic DNA was extracted, and the number of integrated lentiviral copies was quantified by RT-PCR, and the titer was calculated as described previously [51, 52].

### Generation of overexpressing cell lines

1 × 10^5^ HEK293T cells were transduced with 2 × 10⁶ TU lentiviruses encoding either a control lentiviral vector or lentiviral vectors encoding human IL-12, CD19, CD22, or IL-2 at a multiplicity of infection (MOI) of 20. To obtain cell lines stably expressing IL-12, CD19, CD22, and IL-2 respectively, HEK293T cells were sorted for stable expression of corresponding surface proteins using a MoFlo XDP flow cytometer (Beckman Coulter). To obtain CD19/IL-12, CD22/IL-12 or CD19/IL-2 coexpressed HEK293T cell lines, IL-12 or IL-2 expressing HEK293T cells were transduced with lentiviral vectors encoding either a control lentiviral vector or lentiviral vectors encoding human CD19 or CD22 at a MOI of 20 and sorted again.

### Isolation and characterization of EVs

EVs were isolated from HEK293T cells and engineered HEK293T cell lines using previously described methods [26, 28]. 1 × 10^7^ EV producer cell lines were plated in 15 cm dishes and incubated in 25mL serum-free medium for 24 h to allow EVs secretion. Subsequently, the cells were exposed to ultraviolet B (UVB) irradiation (300 J/m²) for 1.5 h. The conditioned medium was collected and centrifuged at 500 × g for 10 min to remove cell debris. Subsequently, the supernatant was filtered through a 0.45-µm sterile filter (Thermo Scientific), and further centrifuged at 14,000 × g for 1 h at 4 °C using an Avanti J-26 S XPI high-performance centrifuge (Beckman Coulter). The isolated EVs were resuspended in 100 µL of phosphate-buffered saline (PBS) and stored at -80 °C until further analysis.

The protein concentration of isolated EVs was determined using a BCA protein analysis kit (Beyotime, China). The concentration and size distribution of EVs were determined by nanoparticle tracking analysis (NTA) based on their Brownian motion. The NTA results were analyzed using the ZetaView multiple-parameter particle tracking analyzer (Particle Metrix), and size distribution data and particle tracking videos were subsequently processed with ZetaView software. EVs characterization by NTA and protein quantification was performed using two independent EVs batches, each measured in three technical replicates. The morphology of EVs was determined by transmission electron microscopy (TEM) using a Tecnai G2 20 TWIN microscope operated at 80 kV. The presence of the EV-associated marker Annexin A1 was further confirmed by immunoblotting, as described below. The concentrations of human IL-12 and IL-2 in EVs were measured using quantitative ELISA kits specific for each cytokine (QuantiCyto, Shenzhen, China).

### EV labeling and data acquisition

Purified EVs were stained with PE-conjugated anti-human IL-12 p70 (clone: 20C2; BD Biosciences) and APC-conjugated anti-human CD19 (clone: HIB19; BioLegend) diluted in PBS filtered through membranes with 0.22 μm-sized pores at 4 °C for 30 min in the dark, washed with filtered PBS and resuspended in filtered PBS. The samples were immediately subjected to flow cytometry (ImageStreamxMkII cytometer, Amnis), and the data were analyzed using IDEAS 6.2 software (Luminex) as described previously [53, 54]. The fluidics settings were set to “low speed/high sensitivity” for all sample data acquisition at 60x magnification.

The same gating strategy was used when different samples were analyzed. The controls included unstained samples, buffer-only controls, and buffer plus reagent controls, and single-stained samples were used for fluorescence compensation. The low-SSC region was circled by side scatter (Ch06) and then used to further analyze the fluorescence events (> 10,000 events were acquired). On the basis of these single-positive fluorescent populations, single-positive gating areas were established, and double-positive gates were set on the basis of the boundaries of the single-positive gates. The low ends of the various gates were defined via unstained samples.

### Western blotting

To characterize the molecules present in EVs and cells, western blotting was conducted. Briefly, purified EVs or cells were lysed with RIPA lysis buffer supplemented with 1 mM PMSF and a protease inhibitor cocktail. Protein lysates (40 µg per sample) were then separated via 4–12% SDS‒PAGE. The resulting proteins were transferred onto PVDF membranes (Millipore, USA) and blocked with 5% skim milk for 1 h at 25 °C. For EV molecule characterization, the membranes were incubated overnight at 4 °C with the following antibodies on a shaker: an anti-Annexin A1 antibody (Abcam, ab214486, 1:2000), and anti-IL-12 p70 antibody (R&D Systems, MAB219-SP, 1:1000). Total STAT4 and phosphorylated STAT4 in T cells were detected using an anti-Stat4 (C46B10) antibody (CST, 2653 S, 1:1000) and an anti-phospho-Stat4 (Tyr693) antibody (CST, 5267 S, 1:1000). After three washes with TBST, the membranes were incubated with HRP-conjugated secondary antibodies (1:1000) at room temperature for 1 h. The membranes were then developed using Pierce ECL reagent (Thermo Fisher, USA), and all images were captured using Image Lab software (Bio-Rad).

### Generation of CAR-T cells

Peripheral blood mononuclear cells (PBMCs) were harvested from whole blood using Ficoll-Paque Plus (GE Healthcare, USA), and CD3 + T cells were isolated from human PBMCs using Miltenyi Biotec CD3 microbeads (Germany). Primary T cells were stimulated with T-Activator CD3/CD28 magnetic beads (Thermo Fisher Scientific, 11131D) at a bead: cell ratio of 1:1 and cultured in CTS medium (Gibco, USA) supplemented with 2% human FBS, 2 mM l-glutamine (Gibco, USA), and 200 IU/mL human recombinant IL-2 (PeproTech, USA). After 24 h, activated T cells were transfected with the anti-CD19 CAR virus or anti-CD22 CAR virus, while non-transduced T cells served as a negative control. Throughout the cultivation process, T cells and CAR-T cells were cultivated at a density of 0.5–2.0 × 10^6^/mL, and the medium was changed every 2–3 days. CAR transduction efficiency was routinely assessed by flow cytometry prior to every functional assay.

### Primary NK cells isolation and culture

Human primary NK cells were obtained from Milestone Biological Science&Technology Co., Ltd. (Shanghai, China). NK cells were isolated by Positive Selection using an NK cell isolation kit (STEMCELL Technologies, Catalog #17855), following the manufacturer’ s protocol. Fresh NK cells were activated using m21-K562 feeder cells (Hangzhou Zhongying Biomedical Technologies, China) at a 1:2 ratio. The NK cells were cultured in complete KBM581 medium (Corning, New York, USA), supplemented with 10% FBS and 200 IU/ mL IL-2 for 10 days prior to use.

### Flow cytometry

For cell surface staining, the cells were resuspended in PBS supplemented with antibodies for 15 min at room temperature. Flow cytometry analysis was conducted using either a NovoCyte flow cytometer (ACEA Biosciences) or a MACSQuant Analyzer 10 flow cytometer (Miltenyi Biotec), and the data were analyzed with NovoExpress software (ACEA Biosciences) or FlowJo software (Tree Star).

PE-conjugated anti-human IL-12 p70 (clone: 20C2; BD Biosciences) and APC-conjugated anti-human CD19 antibodies (clone: HIB19; BioLegend) were used to assess the expression of CD19 and IL-12 on 293T cells. PE-conjugated anti-human IL-2(clone: MQ1-17H12; BioLegend) and APC-conjugated anti-human CD19 antibodies (clone: HIB19; BioLegend) were used to assess the expression of CD19 and IL-2 on 293T cells. For the assessment of CD22 and interleukin-12 (IL-12) expression, PE-conjugated anti-human IL-12 p70 antibody (clone 20C2; BD Biosciences) and Brilliant Violet 421 (BV421)-conjugated anti-human CD22 antibody (clone S-HCL-1; BioLegend) were applied. The following antibodies were used to characterize the phenotype and function of CAR-T cells: PE/Cyanine7-conjugated anti-human CD8 (clone: RPA-T8, BioLegend), BV421-conjugated anti-human CD4 (clone: RPA-T4; BD Biosciences), FITC-conjugated anti-human CD3 (clone: HIT3a; BD Biosciences), APC-conjugated anti-human CD45RO (clone: UCHL1, BioLegend), BV605-conjugated anti-human CD62L (clone: DREG-56; BD Biosciences), BV785-conjugated anti-human PD-1 (clone: EH12.2H7, BioLegend), BV650-conjugated anti-human LAG-3 (clone: HC3C65, BioLegend), PE-conjugated anti-human TIGIT (clone: A15153G; BioLegend), BV421-conjugated anti-human TIM-3(clone: A18087E; Bioligand), and PerCPCy5.5-conjugated anti-human CD4 (clone: RPA-T4; BioLegend) antibodies.

For CD19 CAR expression assays, cells were stained with PE-labeled (Acro Biosystems, Cat.CD9-HP2H3) or FITC-labeled human CD19 protein (Acro Biosystems, Cat. CD9-HP2H3) or APC-conjugated anti‐human EGFR (clone: AY13; BioLegend) For CD22 CAR expression analysis, cells were stained with FITC-labeled (Acro Biosystems, Cat. No. CD2-HF254) or APC-labeled recombinant human CD22 protein (Acro Biosystems, Cat. No. SI2-HA2H4).

### T cell degranulation assay

CAR-T cells (5 × 10^4^ CAR + cells) were co-incubated with Raji cells at an effector: tumor (E: T) ratio of 1:1 in 200 µL RPMI 1640 medium containing APC-conjugated CD107a antibody (clone H4A3; BioLegend) for 4 h. During the incubation, cells were treated with EVs (total protein concentration: 167 µg/mL, approximately 3.4 × 10^11^ EVs particles/mL, IL-12 concentration for EVs containing IL-12: 667 pg/mL) or PBS. After incubation, cells were washed twice with PBS, stained with anti-human CD3/CD8/CAR antibodies, and analyzed by flow cytometry.

### Functional analysis of CAR-T cells

For the cytokine release assays, CAR-T cells (5 × 10^4^ CAR + cells) were seeded in a 96-well plate and cocultured with 5 × 10^4^ Raji cells or K562 cells at an E: T ratio of 1:1 in the presence of various types of EVs (167 µg/mL) or recombinant IL-12 (667 pg/mL). After 24 h, culture supernatants were harvested by centrifugation at 300 × g for 10 min and stored at − 80 °C. Secretion of IFN-γ, TNF-α, and IL-2 in the supernatants were quantified using cytokine-specific ELISA kits (Neobioscience Technology, China).

For the phenotype assay, 3 × 10^5^ CAR-T cells were incubated with 300 µL EVs (167 µg/mL) for 7 days, after which differentiation status and exhaustion marker expression were assessed by flow cytometry.

To analyse the proliferation of T cells, 3 × 10^5^ CAR-T cells were labeled in advance with 2 µM CellTrace™ Violet (Thermo Fisher) and mixed with various types of EVs (167 µg/mL) in 300 µL medium. As a control, labeled CAR-T cells were cultured in presence of 20 ng/mL rhIL-2. After a 96-hour incubation period, cell proliferation was evaluated by flow cytometry.

### In vitro cytotoxicity assay

Raji or K562 cells were labeled with 1 µM CellTrace™ Violet (Thermo Fisher) before being washed with PBS to remove excess dye. 5 × 10^4^ CAR-T cells were subsequently cocultured with 5 × 10^4^ CFSE-labeled Raji or K562 cells at an effector: tumor (E: T) ratio of 1:1 in the presence of various types of EVs (167 µg/mL). The cells were harvested after 24 h and stained with propidium iodide (PI) (BD Pharmingen). Flow cytometry was used to analyze the relative cytotoxicity of CAR-T cells toward tumor cells by quantifying the percentage of dead tumor cells among the CFSE-labeled tumor cells.

### Repeated stimulation assays with EVs

CAR-T cells (3 × 10⁵ CAR + cells) were co-cultured with 1 × 10⁵ Raji cells at a 3:1 ratio of in the presence of EVs (167 µg /mL). After 3 days, T cells were harvested, counted, and re-plated with fresh Raji cells at the same effector-to-target ratio, together with the corresponding EVs. This cycle of counting, replating with fresh tumor cells, and EVs supplementation was repeated every 3 days. At the end of each stimulation round, T cells were analyzed by flow cytometry to assess T cell subsets and expression of exhaustion markers.

### Intracellular IFN-γ and phosphorylated STAT-4 staining

2 × 10^6^ CAR-T cells were resuspended in 2 mL medium and incubated with different EVs (167 µg/mL) or PBS, in the presence of a protein transport inhibitor (BD Bioscience, 54655). Cell surface staining was conducted, followed by cell fixation and permeabilization via a Cytofix/Cytoperm Kit (BD Biosciences, 558050). The cells were subsequently stained with the intracellular antibody FITC-conjugated anti-human IFN-γ (clone 4 S. B3; BioLegend) at 4 °C for 30 min. After a wash step, flow cytometry analysis was performed.

Before analysis, human NK cells were deprived of IL-2 by incubation in serum-free medium for 24 h. Following starvation, 2 × 10^6^ NK cells were cultured with different EVs (total protein concentration: 250 µg/mL, IL-12 concentration for EVs containing IL-12: 1000 pg/mL) in 2 mL medium for 45 min. Cells were then harvested for surface and intracellular staining. Surface markers were stained first, followed by fixation and permeabilization using the Cytofix/Cytoperm Kit (BD Biosciences, cat. 558050), according to the manufacturer’s protocol. IFN-γ secretion was measured using ELISA kits specific for human IFN-γ (QuantiCyto, Shenzhen, China). Intracellular staining was performed using AF647-conjugated anti-pSTAT4 (Y693) antibody (BioLegend, cat. 562074) at 4 °C for 30 min. After washing, samples were analyzed by flow cytometry.

### Binding and cellular uptake of EVs

EVs were labeled with 10 µM DIO (Invitrogen, USA) in PBS at 4 °C for 2 h, washed twice with PBS to remove free dye and then pelleted by ultracentrifugation at 14,000 × g for 1 h at 4 °C. To analyze the efficiency of EV uptake by CAR-T cells, 3 × 10^5^ CAR-T cells were incubated with 167 µg/mL DIO-labeled EVs in 300 µL medium for 4 h at 37 °C and then analyzed by flow cytometry (Nova Express).

### Imaging of CAR-T-cell–EV interactions

To image and evaluate the interactions between CAR-T cells and EVs, 2 × 10^6^ CAR-T cells were resuspended in 2 mL medium and treated with different EVs (167 µg/mL) for 45 min at 37 °C. The cells were then collected and washed with PBS to remove unbound EVs, followed by staining with anti-EGFR-APC, anti-CD3-FITC, and anti-IL-12p70-PE antibodies at room temperature for 30 min.

The samples were immediately assessed by flow cytometry (ImageStreamxMkII cytometer, Amnis). A minimum of 5000–15,000 cell events were acquired on each sample with a low acquisition rate for high sensitivity at 60x magnification. Compensations were performed with at least 2000 cell events acquired from singly stained tubes. The acquired images and data were analyzed via IDEAS 6.2 software (Luminex) as previously described [55, 56]. The gating strategy employed the following steps: focusing on events based on the gradient RMS values; identifying singlets by evaluating the area of the bright field (Ch01) vs. the aspect ratio of the bright field (Ch01), with a defined aspect ratio > 0.8; further selecting singlets using CD3-FITC (Ch02) to define T cells; within the T-cell gate, distinguishing between CAR-positive and CAR-negative T cells using EGFR-APC (Ch05); and detecting cells bound to EVs by IL-12p70-PE (Ch03) positivity within the CAR-positive T-cell population, as EVs were labeled with IL-12p70. The same gating strategy was applied for the analysis of different samples.

### RNA-seq and analysis

CAR-T cells were treated with control EVs, CD19 EVs, IL-12 EVs, or CD19-IL12 EVs for 24 h. The cells were then harvested and washed with PBS. RNA library sequencing was performed on the Illumina HiSeq2500 platform by Gene Denovo Biotechnology Co., Ltd. (Guangzhou, China). The raw sequencing data in this study are available upon request. The DESeq2 R package was used to estimate differential gene expression. Differentially expressed genes were defined as genes with Q values < 0.05 and log2FC > 1. Pathways of enrichment analysis of differentially expressed genes were analyzed via the clusterProfiler R package. Gene set enrichment analysis (GSEA) was conducted via the GSEA tool in R.

### IL-12 regulon identification and analysis

The IL-12-regulated gene set was obtained by comparing the upregulated DEGs in the IL-12 EV group with those in the control EV group and the upregulated DEGs in the CD19/IL-12 EV group with those in the CD19 EV group (thresholds: log2-fold change > 1, FDR < 0.05; Table S1). Gene signature scores were calculated as described previously [57]. The expression data used for scoring the gene signature included gene expression data from activated anti-CD19 CAR-T-cell products derived from CLL patients published by Joseph A. Fraietta et al. [3]. The enrichment scores of the IL-12 regulon were analyzed via single-sample GSEA (ssGSEA). The Mann‒Whitney U test was performed to compare ssGSEA enrichment scores between responders and nonresponders.

### In vivo mouse experiments

All animal experiments were performed in compliance with the procedures adopted by the Laboratory Animal Welfare and Ethics Committee of Tongji Hospital, Wuhan, China (TJH-202011005).

Female 5- to 6-week-old NCG mice (obtained from GemPharmatech, China) were subcutaneously injected in the right flank with Raji-luc lymphoma cells (5 × 10^6^ cells in 50 µl of Matrigel matrix and 50 µl of PBS). Following tumor implantation, tumor length and width were measured with a digital caliper every 3–5 days, and tumor volume was calculated via the formula (width^2 × length)/2. In addition, weekly in vivo imaging of Raji-luc cells in the mice was performed via an IVIS imaging system, and the obtained data were analyzed using Living Image software. Once the subcutaneous tumors reached 50 to 100 mm^3^, the mice were intravenously injected with 1 × 10^6^ CAR-T cells (with CAR transduction efficiency of 15%) or 6.67 × 10^6^ untransduced T cells as control. The mice were randomly assigned to different treatment groups and received intratumoral injections of control EVs, CD19 EVs, IL-12 EVs, or CD19/IL-12 EVs (with protein content of 200 µg, approximately 4 × 10^11^ EV particles per mouse) on days 1, 3, and 5 following CAR-T-cell treatment. The body weights and overall conditions of the mice were monitored every three days. The mice were euthanized if they exhibited signs such as initial weight loss exceeding 20%, significant lethargy, a hunched posture, severe diarrhea, severe dermatitis, or if the tumor volume exceeded 1500 mm^3^.

To evaluate inflammatory cytokines and monitor CAR-T-cell amplification, 100 µl of peripheral blood from each mouse was collected via orbital blood for subsequent flow cytometry analysis and cytokine assays.

### Droplet digital PCR

The expansion and persistence of CAR-T cells in mice were detected via droplet digital PCR as previously described [58]. The following primers were used in this study: the CAR forward primer 5’-CAGCAAAAA TACGACCTCCTCACT-3’, the reverse primer 5’-TGGTGCTGCCTTTGA TCTCA-3’, and the probe 5’-FAM-TTGGCGGGAGGGACC-3’. The samples were tested via a Quantalife QX200 Droplet Digital PCR system (Bio-Rad). The data were analyzed using QuantaSoft software version 1.7.4 (Bio-Rad).

### Measurement of serum cytokine levels

Mouse serum was isolated from whole blood supernatants for cytokine analysis. The serum levels of human IFN-γ, TNF-α, IL-12p40, IL-2, IL-10, and IL-4 were evaluated by a human cytokine cytometric bead array kit (Human IL-2 Flex Set #cat.558270; Human IL-4 Flex Set #cat.558272; Human TFN-a Flex Set #cat.560112; Human IL-6 Flex Set #cat.558276; Human IL-12 Flex Set #cat.55828; Human IFN-g Flex Set cat.560111; BD Biosciences) according to the manufacturer’s instructions. The data were collected using a NovoCyte Flow Cytometer (ACEA Biosciences) and analyzed using FCAP.GUI. software.

### Histopathological analysis

After therapy, the major organs of the mice were excised, fixed in 4% paraformaldehyde, and embedded in paraffin. Sections were cut at 3 μm, and pathological examination was performed by hematoxylin and eosin (H&E) staining.

### Statistical analysis

All experiments were independently repeated at least three times. Data are presented as mean ± SEM and were analyzed using GraphPad Prism version 9.0.The statistical significance of differences between two groups was determined via two-tailed paired or unpaired t tests, whereas comparisons between multiple groups were performed via one-way or two-way ANOVA. Animal survival was assessed via the log-rank (Mantel‒Cox) test. The error bars in this study represent mean ± standard error of the mean (SEM). A *p* value of less than 0.05 was considered statistically significant, with significance levels indicated by asterisks (ns for not significant; *p < 0.05; **p < 0.01; ***p < 0.001; ****p < 0.0001).

## Supplementary Information

Below is the link to the electronic supplementary material.

Supplementary Material 1

Supplementary Material 2

Supplementary Material 3

Supplementary Material 4

## Data Availability

No datasets were generated or analysed during the current study.
