# Supplementary material for: Improving CAR-T cell function through a targeted cytokine delivery system utilizing car target-modified extracellular vesicles
Source: Exp Hematol Oncol. 2025 Aug 25;14:110. doi: 10.1186/s40164-025-00701-z (PMC12379361; doi:10.1186/s40164-025-00701-z)
Supplement: Supplementary file 2 — Supplementary Material 2 [file 40164_2025_701_MOESM2_ESM.pdf]

Table.S1 The percentages of CAR-T cells in functional assay experiments.

Figure	2B-E, 2G (n=3)			2F (n=5)				
**CAR-T cells (%)**	23.27	25.66	18.67	23.27	18.67	11.05	8.95	9.53
**Figure**	**3D (n=4)**							
**CAR-T cells (%)**	22.10	22.80	22.30	20.30				
**Figure**	**3E (n=7)**							
**CAR-T cells (%)**	14.94	8.87	25.50	14.49	7.39	16.53	6.65	
**Figure**	**3F (n=3)**			**3G (n=4)**				
**CAR-T cells (%)**	22.77	10.00	11.49	48.18	32.38	14.52	14.27	
**Figure**	**3I (n=3)**							
**CAR-T cells (%)**	18.93	12.03	7.00					
