# Supplementary material for: Improving CAR-T cell function through a targeted cytokine delivery system utilizing car target-modified extracellular vesicles
Source: Exp Hematol Oncol. 2025 Aug 25;14:110. doi: 10.1186/s40164-025-00701-z (PMC12379361; doi:10.1186/s40164-025-00701-z)
Supplement: Supplementary file 3 — Supplementary Material 3 [file 40164_2025_701_MOESM3_ESM.docx]

# **Supplementary Figure** and Figure legend


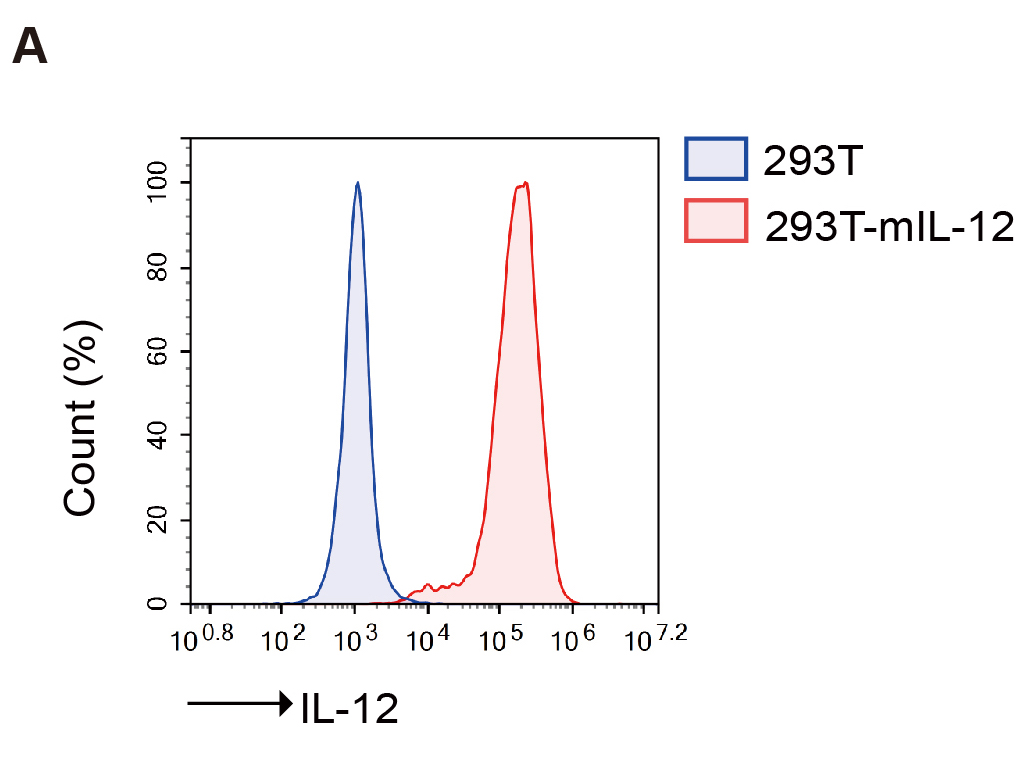


**Supplementary Figure 1**

**
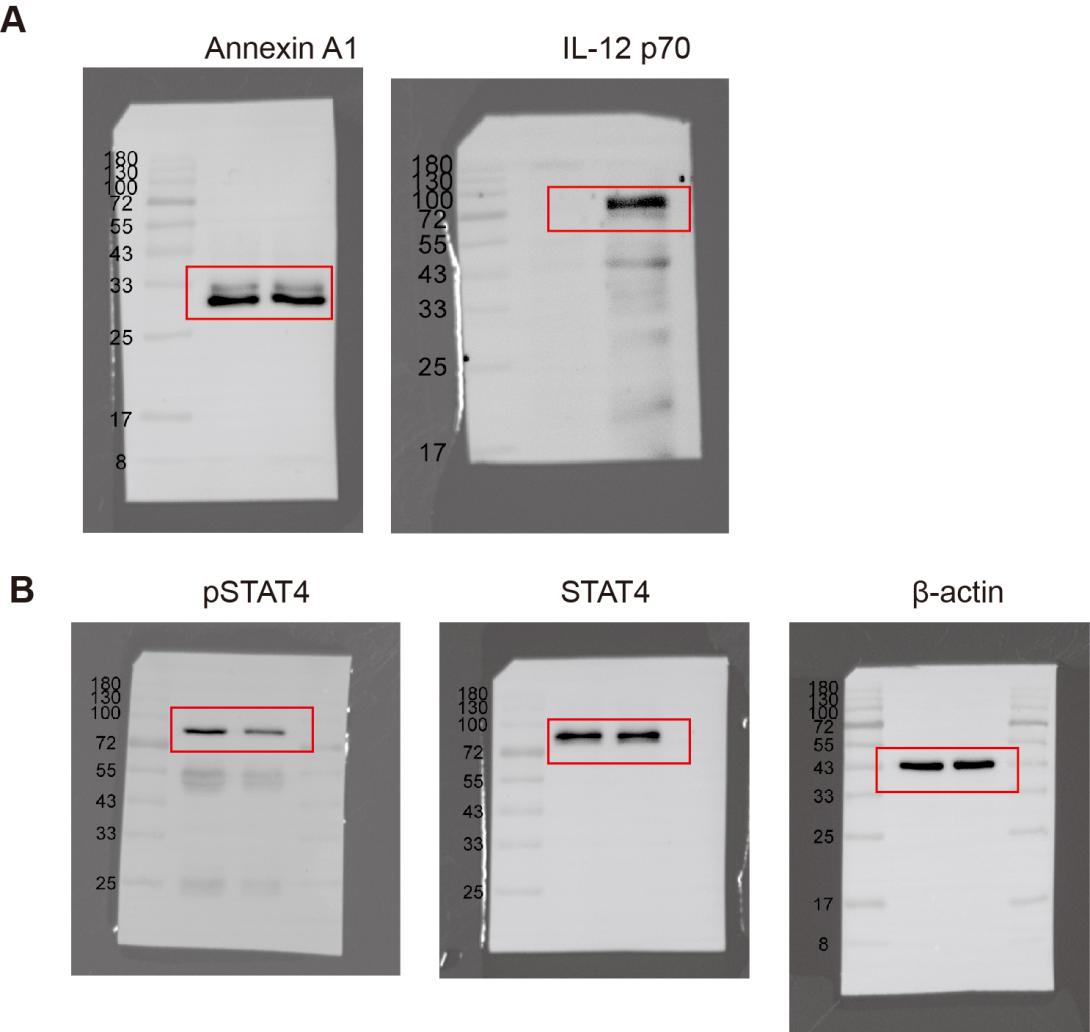
**

**Supplementary Figure 2**

**
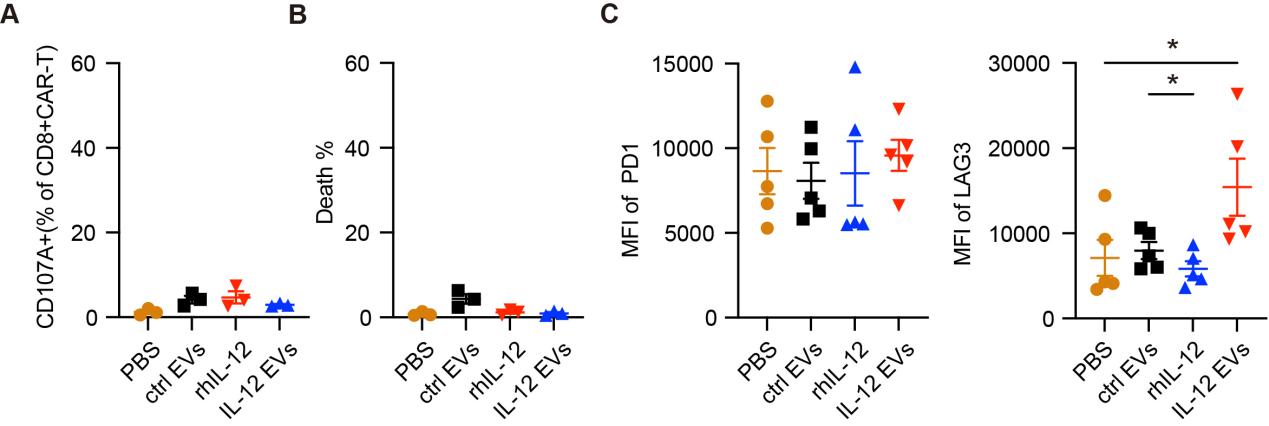
**

**Supplementary Figure 3**

**
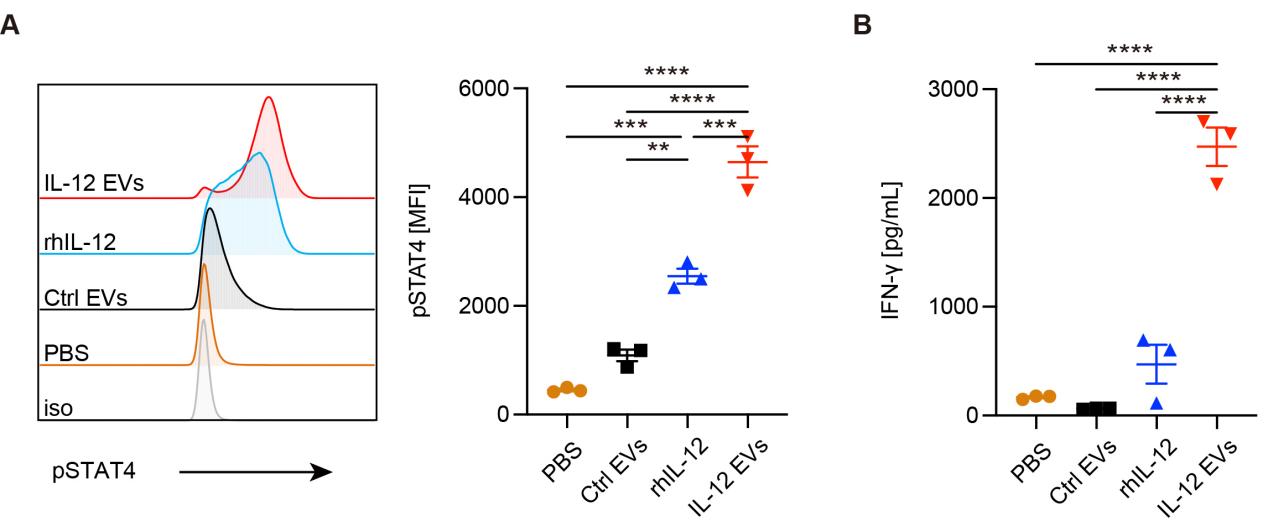
**

**Supplementary Figure 4**

**
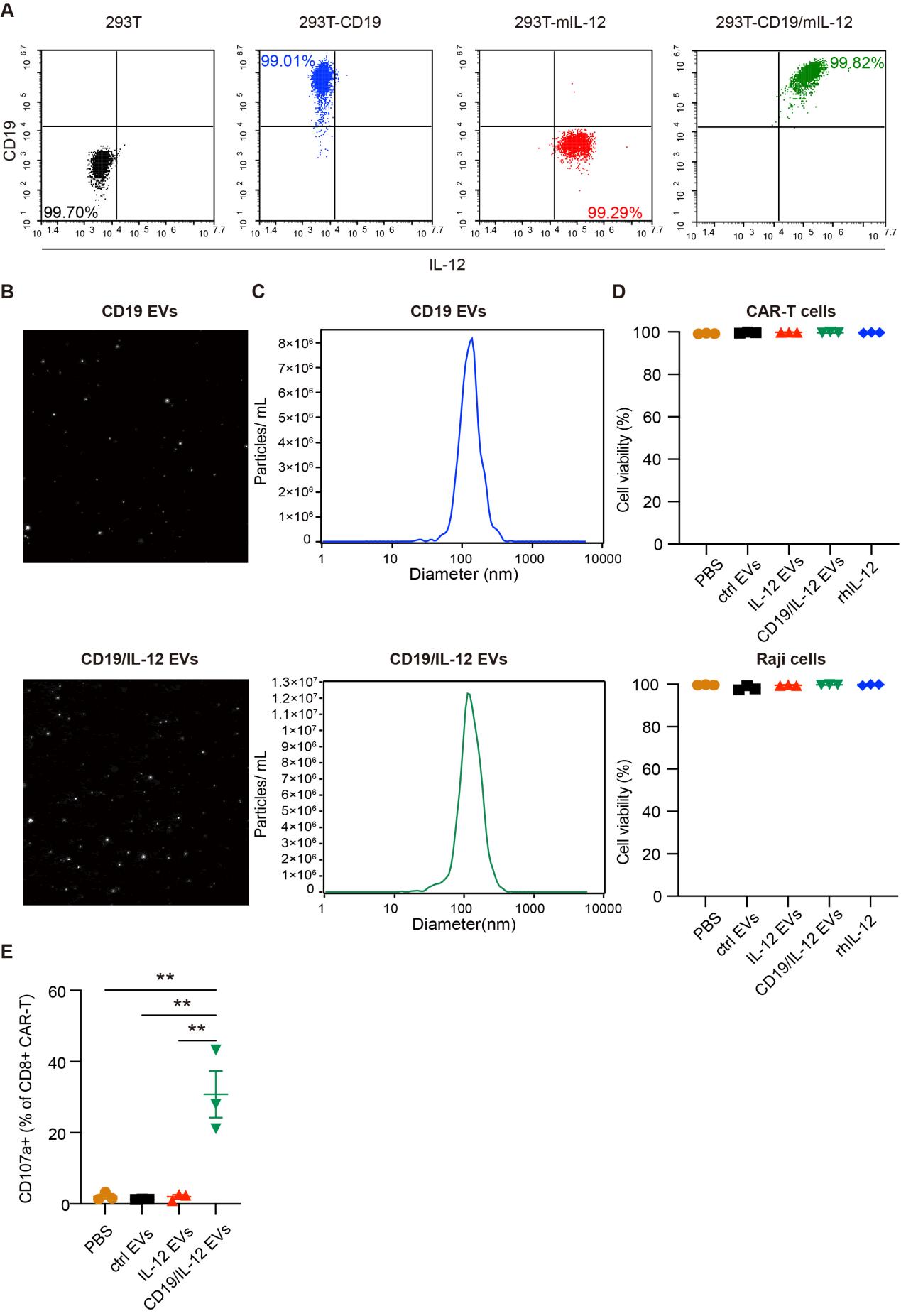
**

**Supplementary Figure 5**

**
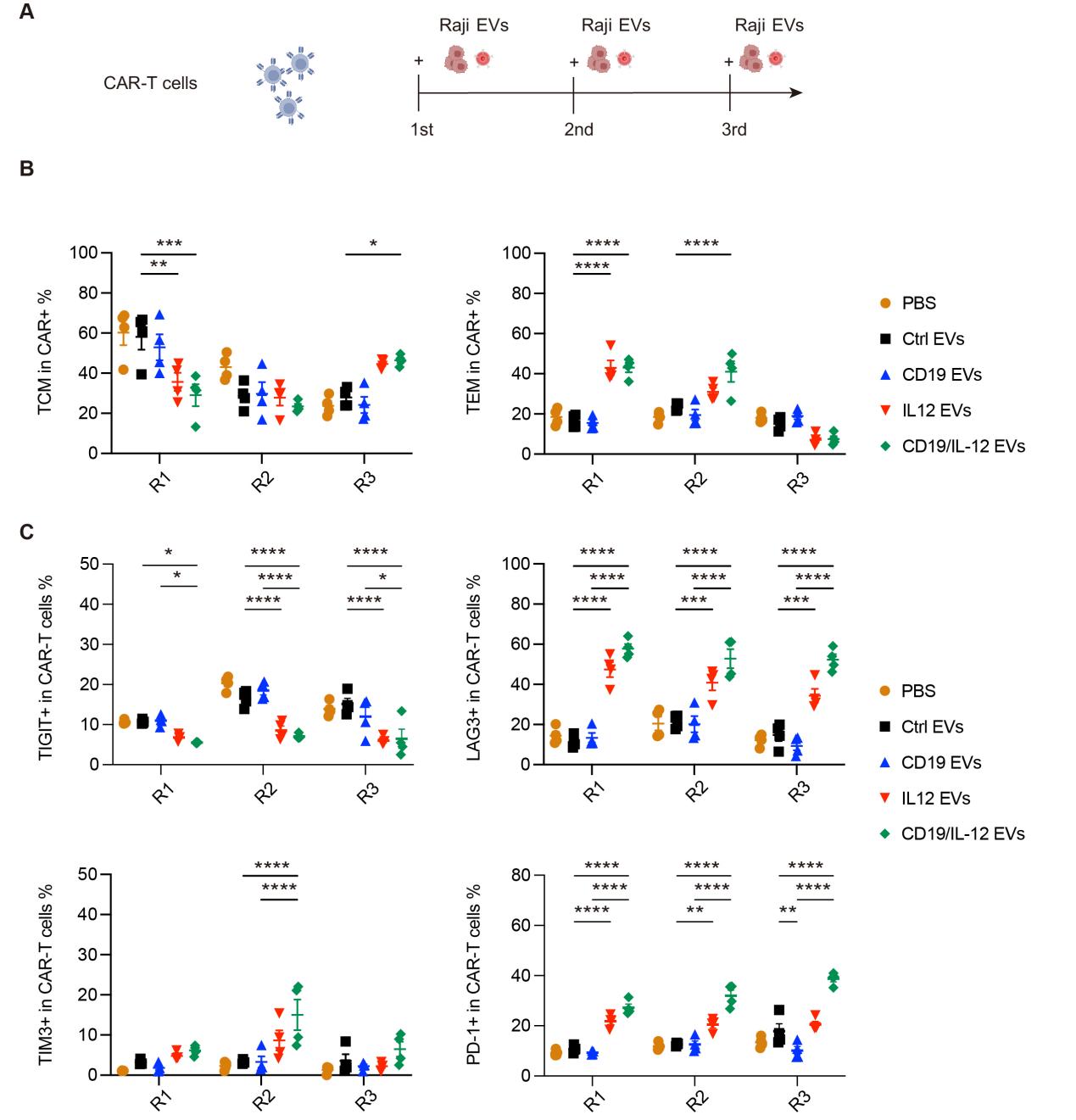
**

**Supplementary Figure 6**

**
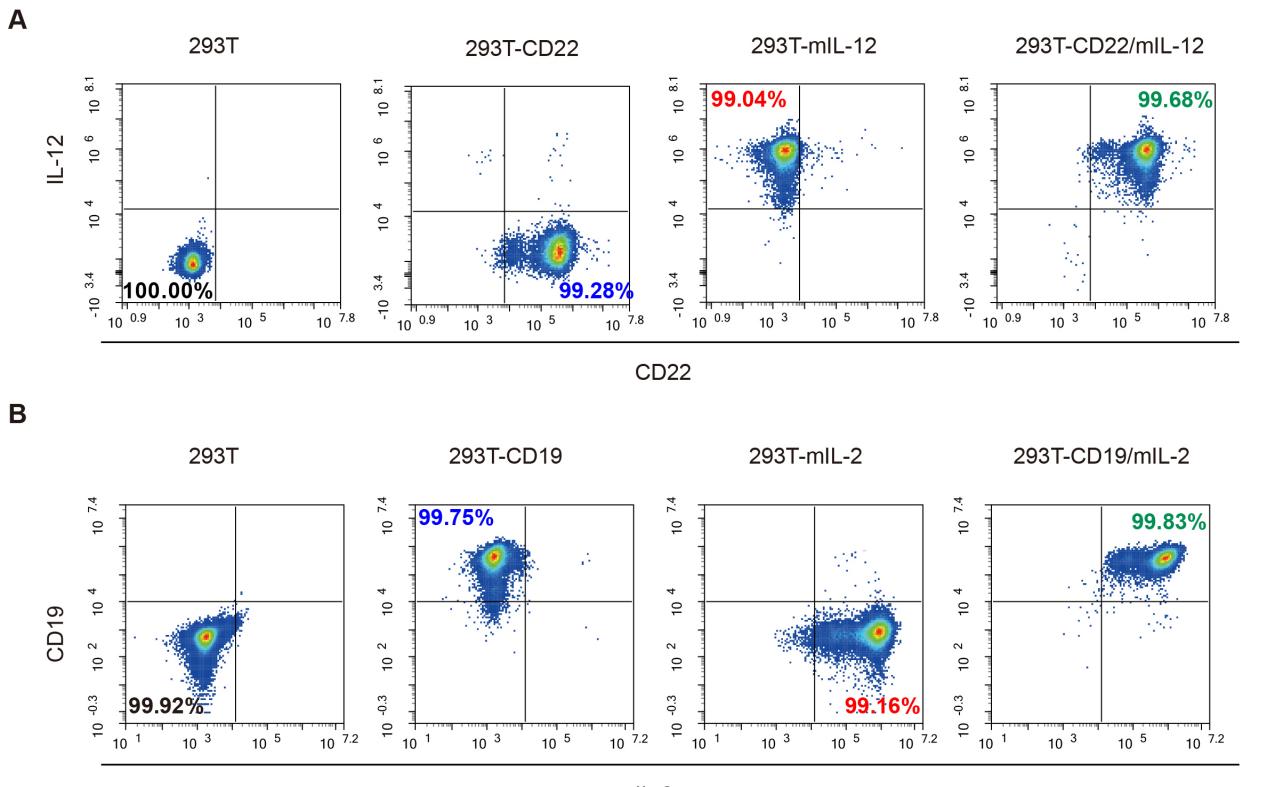
**

**Supplementary Figure 7**

**
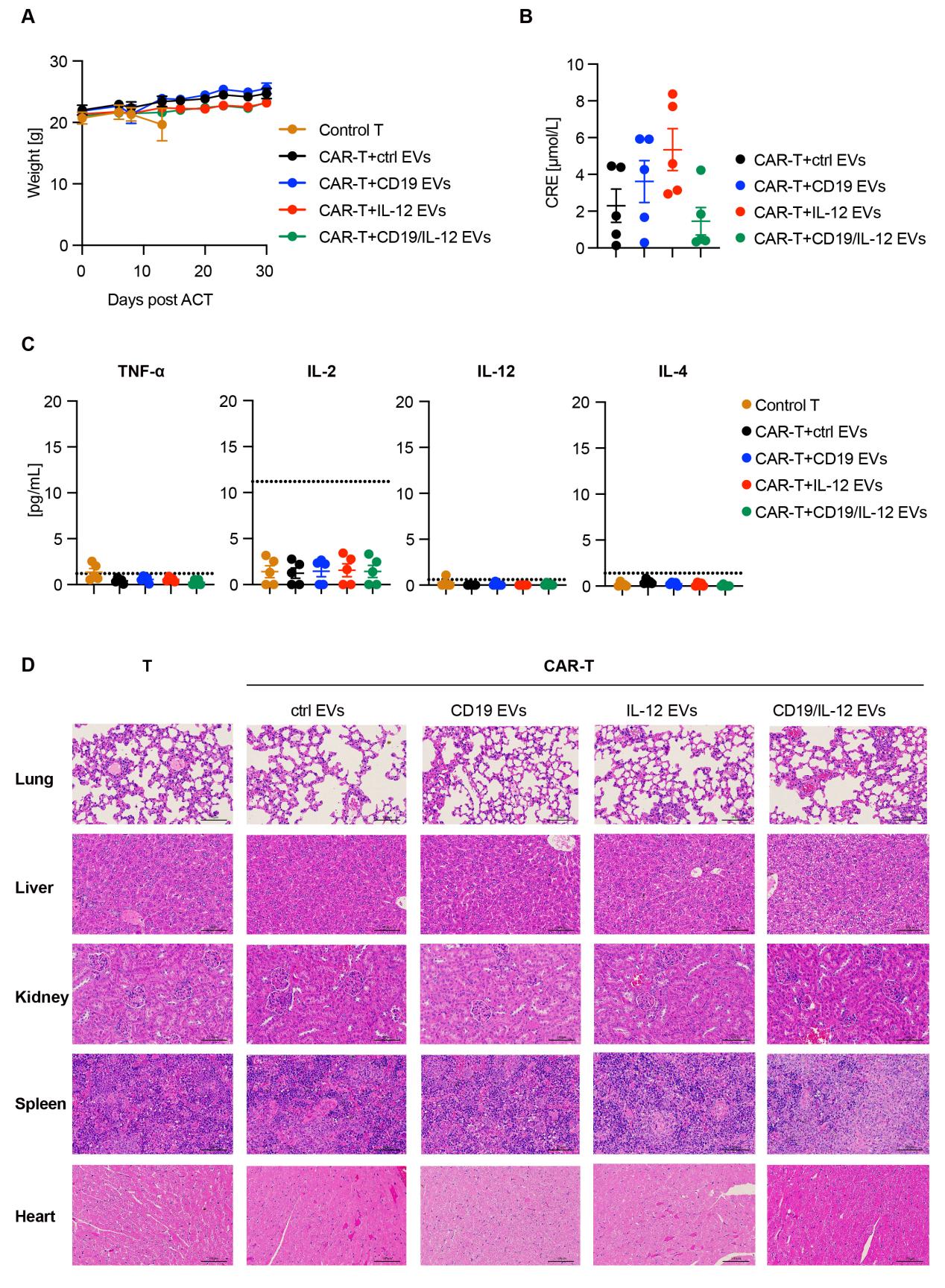
**

**Supplementary Figure 8**

**
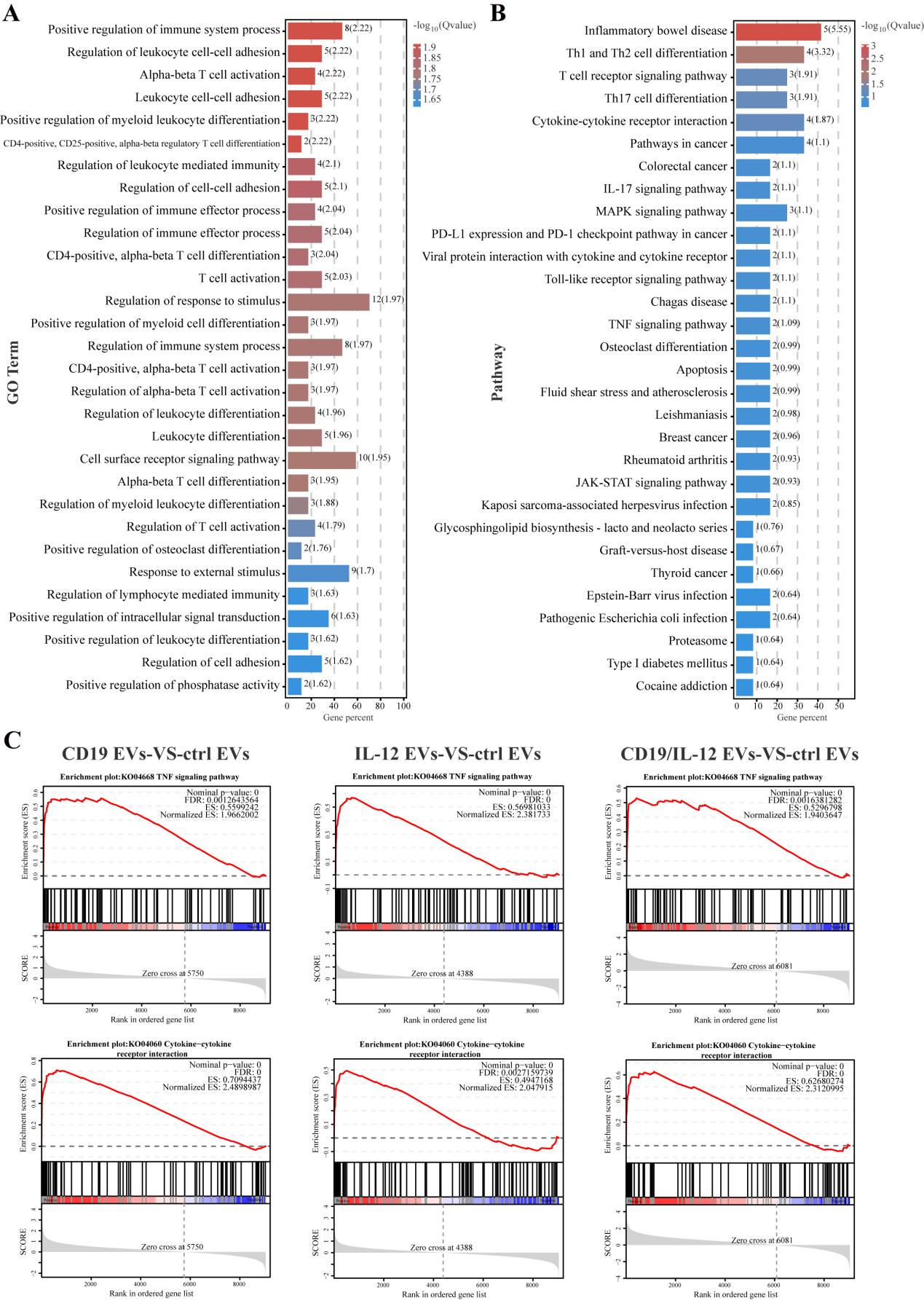
**

**Supplementary Figure9**

# **Supplementary Figure legend**

**Supplementary Figure 1.** Flow cytometry was performed to detect the expression of IL-12 on the surface of HEK293T-mIL-12 cells. Experiments are representative of 3 independent repeats.

**Supplement Figure 2. Uncropped gels for Western Blots**. (A) The original and uncropped gels for Western Blots in Figure 1C. (B) Uncropped gels for Western Blots in Figure 2A. Experiments are representative of 3 independent repeats.

**Supplementary Figure 3.** **Effect of IL-12 EVs on** **CAR-T cells.** (A-B) CAR-T cells were mixed with 5×10^4^ K562 cells at an effector-to-target ratio of 1:1 and treated with PBS, control EVs , rhIL-12 and IL-12 EVs respectively. (A) CD107a expression in CD8+ CAR-T cells was detected by flow cytometry (n = 3 donors). (B) K562 cell death was determined using PI (BD Pharmingen) and analyzed by using flow cytometry after 24 h (n = 3 donors). (C) 3 × 10^5^ CAR-T cells were mixed with Raji cells at an effector-to-target ratio of 1:1 and treated with PBS, control EVs, and rhIL-12 and IL-12 EVs differently. PD-1 and LAG-3 expression in CAR-T cells was analyzed via flow cytometry after 7 days of treatment (n = 5 donors). Data are presented as mean ± SEM. Statistical analysis was performed using one-way ANOVA. **p* <0.05. Protein concentration of EVs is 167μg/mL, and rhIL-12 concentration is 667 pg/mL.

**Supplement Figure 4. IL-12 EVs enhance STAT4 signaling and IFN-γ secretion in primary human NK cells.** (A) Purified human peripheral blood NK cells were stimulated under the indicated conditions for 45 minutes. Intracellular pSTAT4 (Y693) levels were assessed by flow cytometry following stimulation; (B) IFN-γ production of NK cells after cultured with PBS, Ctrl EVs, IL-12 EVs and recombinant IL-12 (1 ng/mL) for 24 hours (n = 3 donors). Protein concentration of EVs is 167μg/mL, and rhIL-12 concentration is 1000 pg/mL.

**Supplementary Figure 5.** **Production and characterization of CD19 EVs and CD19/IL-12 EVs.** (A) Flow cytometry detection of CD19 and IL-12 p70 expression in EV-derived HEK293T cell lines (independent experiments with n = 3). (B, C) NTA was used to examine the Brownian motion and size distribution of CD19 EVs and CD19/IL-12 EVs (independent experiments with n = 3). (D) The cell viability of CAR-T cells and Raji cells treated by different EVs or recombinant IL-12 (independent experiments with n = 3). (E) The degranulation of CAR-T cells stimulated by different EVs without tumor cells. Statistical analysis was performed using one-way ANOVA. **p* <0.05. (independent experiments with n = 3). Protein concentration of EVs is 167μg/mL, and rhIL-12 concentration is 667 pg/mL.

**Supplement Figure 6. Long-term effects of EV treatments on CAR-T cells**. (A) Schematic diagram of repeated EV stimulation. 3 × 10⁵ CAR-T cells (cell density: 1*10^6^/mL) were co-cultured with Raji tumor cells at an effector-to-target ratio of 3:1 and treated with PBS, control EVs , CD19 EVs, or IL-12–loaded EVs as indicated. Fresh medium, EVs, and tumor cells were replenished every 3 days. (B) Flow cytometric analysis of central memory and effector memory T cell subsets in CAR-T cells following each round of stimulation. (C) Continuous evaluation of exhaustion marker expression (TIM-3, LAG-3, PD-1, and TIGIT) in CAR-T cells following each round of stimulation (n = 4 donors). Mean ±SEM. **p* <0.05. Protein concentration of EVs is 167μg/mL, and rhIL-12 concentration is 667 pg/mL.

**Supplement Figure 7.** (A) Flow cytometry detection of CD22 and IL-12 p70 expression in EV-derived HEK293T cell lines. (B) Flow cytometric analysis of membrane-bound IL-2 expression on HEK293T-mIL-2 cells. Experiments are representative of 3 independent repeats.

**Supplementary Figure 8. Safety of** **intratumoral administration of CD19/IL-12 EV**s. (A) Body weights of the mice during the time course of the various treatments. (B) Renal function (creatinine, CRE) of the mice was determined via chemical tests on day 14 after ACT. (C) Cytokine serum levels (TNF-α, IL-2, IL-12 and IL-6) in the mice were quantified via a CBA kit on day 9 after ACT. (D) Representative images of H&E-stained vital organs on day 30 after ACT. Scale bars, 50 μm. A dashed line is indicative of the lower detection limits. Data are presented as mean ± SEM. Statistical analysis was performed using one-way ANOVA (n = 5 mice per group).

**Supplementary Figure 9. Effects of CD19 EVs, IL-12 EVs and CD19/IL-12 EVs on the transcriptome of CAR-T cells.** (A, B) The top 30 most enriched pathways identified by (A) GO pathway analysis and (B) KEGG pathway analysis of DEGs that shared the same expression pattern between the control EVs versus IL-12 EVs and between the CD19 EVs versus CD19/IL-12 EVs. Count: Number of genes related to the enriched GO or KEGG pathway. The color of the bar denotes the -log10 (*p* value). (C) GSEA plots of the TNF signaling pathway and cytokine-cytokine receptor interaction in control EVs versus CD19/IL-12 EVs. The normalized enrichment score (NES) and statistical significance/false discovery rate (FDR) Q value are indicated.
