# Supplementary material for: Improving CAR-T cell function through a targeted cytokine delivery system utilizing car target-modified extracellular vesicles
Source: Exp Hematol Oncol. 2025 Aug 25;14:110. doi: 10.1186/s40164-025-00701-z (PMC12379361; doi:10.1186/s40164-025-00701-z)
Supplement: Supplementary file 4 — Supplementary Material 4 [file 40164_2025_701_MOESM4_ESM.zip › NTA data/Ctrl EVs.pdf]

Electrophoresis & Brownian Motion
Video Analysis
Laser Scattering Microscopy

Operator (Report): Administrator
Video Operator: Administrator

Sample Parameters
Sample Name: 293
Comment: Sample Remarks0:
Sample Remarks1:
Sample Remarks2:
Electrolyte: PBS
Temperature: 20.83 °C sensed
pH 7.0 entered
Conductivity: 10.71 µS/cm entered

Result (sizes in nm)

	Number	Concentration	Volume
Median (X50)	133.6	133.6	195.2
Span	55.7	55.7	81.5

Concentration: 1.1E+8 Particles / mL
Dilution Factor: 4000
Original Concentration: 4.2E+11 Particles / mL

Quality
Average Counted Particles per Frame: 220
Number of Traced Particles: 1941

Measurement Parameters
Cell S/N: NTA

Measurement Mode: Size Distribution 3 Cycles
11 Positions

Analysis Parameters
Max Area: 1000, Min Area: 5, Min Brightness: 20

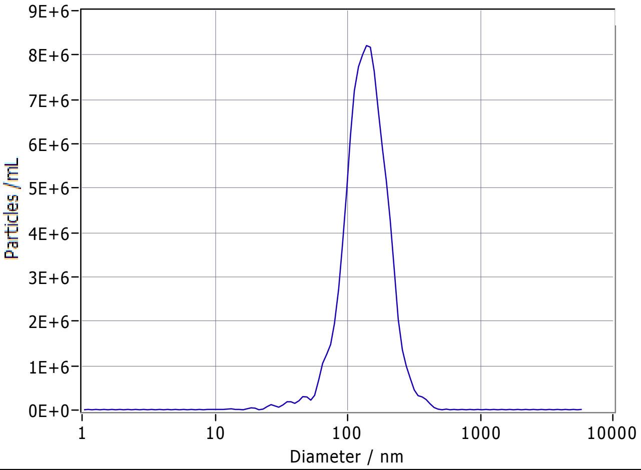

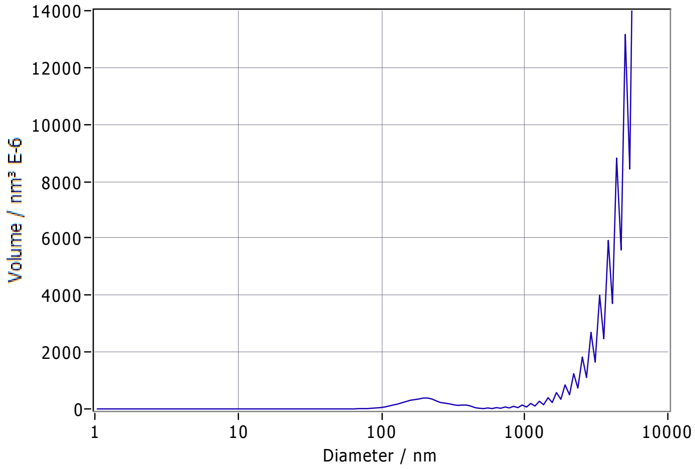

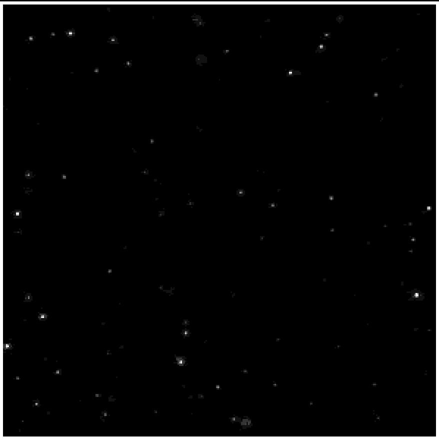

Peak Analysis (Concentration)

Diameter / nm	Particles/mL	FWHM / nm	Percentage
138.9	8.2E+6	118.8	99.4
13.0	2.6E+4	3.0	0.1
8.7	1.1E+4	1.1	0.0
6.6	1.0E+4	5.7	0.1
1.7	9.7E+3	1.1	0.1

X Values

	Number	Concentration	Volume
X10	83.4	83.4	123.8
X50	133.6	133.6	195.2
X90	207.7	207.7	333.4
Span	0.9	0.9	1.1
Mean	146.1	146.1	218.1
StdDev	55.7	55.7	81.5

Comment

(Signature)

Analyzed Video: D:\NTA date\20221207\20221207_0002_293_size.avi

ZetaVIEW S/N 17-310, Software ZetaView 8.04.02, Camera 0.743 µm/px

Experiment: 2022-12-07 09:52, Report: 2022-12-07 09:59
